# Pharmacological inhibition of SPAK-NKCC1 complex attenuates astrogliosis and restores cerebral blood flow in a mouse model of VCID

**DOI:** 10.1186/s40478-025-02137-2

**Published:** 2025-10-24

**Authors:** Khadija Habib, Md. Tipu Sultan, Israt Jahan, Md. Shamim Rahman, Sujan Kumar Kundu, Barnali Sarker, Rabia Islam, Tanvir Ahmed, Ian A. Mendez, Guodong Cao, Dandan Sun, Vesna Tesic, Mohammad Iqbal H. Bhuiyan

**Affiliations:** 1 Department of Neurology, Louisiana State University Health Sciences Center, Shreveport, LA 71103 USA; 2 Department of Pharmaceutical Sciences, University of Texas at El Paso, El Paso, TX 79968 USA; 3 Nostrum Hospital, Dhaka, Bangladesh; 4 Department of Neurology, University of Pittsburgh, Pittsburgh, PA 15213 USA; 5 Veterans Affairs Pittsburgh Health Care System, Geriatric Research Education and Clinical Center, Pittsburgh, PA 15240 USA; 6 Institute for Cerebrovascular and Neuroregeneration Research (ICNR), Louisiana State University Health Sciences Center, Shreveport, LA 71103 USA; 7 Department of Pathology & Translational Pathobiology, Louisiana State University Health Sciences Center, Shreveport, LA 71103 USA

**Keywords:** VCID, BCAS, BBB integrity, Astrogliosis, ZT-1a, CBF

## Abstract

Vascular contributions to cognitive impairment and dementia (VCID) are one of the leading causes of dementia, where reactive astrogliosis, blood-brain barrier (BBB) disruption, and white matter lesions (WML) are the key features. However, the molecular and cellular mechanisms underlying VCID are not well understood. Na-K-Cl cotransporter 1 (NKCC1) activation via its upstream regulatory kinase SPAK (STE20/SPS1-related proline/alanine-rich kinase) causes intracellular Na^+^ overload, hypertrophy, and astrogliosis, a cascade that has been implicated in VCID. In this study, we investigated whether treatment with the SPAK inhibitor ZT-1a at the symptomatic stage in a VCID mouse model is effective in reducing reactive astrogliosis and BBB breakdown, and in improving cerebral blood flow (CBF). VCID was induced in adult C57BL/6J mice using bilateral carotid artery stenosis (BCAS), and either Vehicle (Veh, DMSO) or ZT-1a was administered from weeks 4 to 8 post-BCAS. CBF was monitored by laser speckle imaging, and cognitive deficits were assessed using the Morris water maze test. BBB integrity, astrocytic endfeet coverage, and demyelination were assessed by immunofluorescence (IF) analysis. BCAS mice exhibited a biphasic reduction of CBF and cognitive impairments, parallel with a significant loss of myelin basic protein (MBP) in white matter tracts. Increased expression and phosphorylation of NKCC1 were detected in GFAP^+^ astrocytes and Iba1^+^ microglia/macrophage following BCAS. Reduced ZO-1, Claudin-5, and AQP4 expression in vessels and extravasation of serum albumin into the brain parenchyma in BCAS mice indicate the loss of BBB integrity. Importantly, ZT-1a treatment of the BCAS mice significantly improved CBF recovery and prevented the learning and memory deficit. These mice displayed reduced astrogliosis, microglial activation, MMP-2/9 expression, BBB damage, and axonal demyelination. Our results strongly suggest that hypoperfusion-induced SPAK-NKCC1 activation contributes to reactive astrogliosis, BBB disruption, CBF reduction, and cognitive impairment. The SPAK-NKCC1 complex represents a modifiable therapeutic target for counteracting VCID progression.

**Supplementary Information:**

The online version contains supplementary material available at 10.1186/s40478-025-02137-2.

## Introduction

Vascular contributions to cognitive impairment and dementia (VCID) are among the leading causes of dementia after Alzheimer’s disease (AD), particularly in the aging population [23]. VCID encompasses a spectrum of cognitive impairments, from mild cognitive decline to dementia, and is emerging as a significant public health concern worldwide [59, 81]. Advances in healthcare have led to a rapidly growing population, suggesting that the prevalence of dementia-related disorders, including VCID, will continue to rise. Since there are no FDA-approved drugs for VCID treatment, the development of effective therapies is urgently required. Recent clinical studies have highlighted chronic cerebral hypoperfusion (CCH) as a major driver of vascular pathology and the clinical manifestations of VCID [56]. Although the underlying mechanisms and related disease processes of VCID are not well understood, blood-brain barrier (BBB) disruption, reactive astrogliosis, and demyelination have been identified as hallmarks of VCID [9, 53, 56].

Recent studies have identified BBB breakdown as an early biomarker of human cognitive dysfunction [13]. The tight junctions (TJ) of the BBB function as a selective barrier between the bloodstream and the brain. The TJ consists of three integral membrane proteins, namely, claudin, occludin, and junction adhesion molecules, as well as several cytoplasmic accessory proteins, including Zonula occludens (ZO-1) [32, 52]. Astrocytic endfeet also support BBB integrity and function by ensheathing blood vessels and stabilizing TJs between endothelial cells. Loss of astrocytic endfeet, along with BBB disruption, has been associated with reduced cerebral blood flow (CBF) and impaired hemodynamic responses in stroke and AD [26, 80]. However, whether astrocytic endfeet loss contributes to CBF regulation in VCID remains unknown.

Na^+^-K^+^-2Cl^−^ cotransporter isoform 1 (NKCC1), together with its upstream kinase SPAK (STE20/SPS1-related proline/alanine-rich kinase), play a critical role in the pathology of numerous brain diseases, including VCID [24]. Previously, we and others demonstrated that NKCC1 activity causes intracellular Na^+^ overload in astrocytes, leading to hypertrophy, swelling and astrogliosis following in vitro or in vivo ischemia [34, 65, 68]. SPAK-mediated activation of NKCC1 has also been implicated in exacerbating post-ischemic BBB damage and brain edema [8, 75]. Recently, we reported that administering the non-ATP-competitive specific inhibitor of SPAK kinase ZT-1a during the early stage of VCID (2–4 weeks after bilateral carotid artery stenosis (BCAS) procedure) in mice prevented reactive astrogliosis, demyelination, and memory impairment [9]. However, it remains unknown whether ZT-1a treatment is as effective at the symptomatic stage of VCID (4–8 weeks post-BCAS) as it is at the early stage, and whether it can restore CBF at the advanced stage.

In this study, we report that BCAS induces a biphasic reduction of CBF, disrupts the BBB, and impairs cognitive function in C57BL/6J mice, parallel with a marked loss of myelin basic protein (MBP) in white matter tracts and neuronal death in the hippocampus (HP). Most importantly, treatment with ZT-1a during the symptomatic stage of VCID significantly restored CBF, reversed these pathologies, and improved cognitive function, highlighting the therapeutic potential of ZT-1a for VCID.

## Materials and methods

### Animals and microcoil BCAS model

A total of 134 male C57BL/6J mice used in this study were obtained from Jackson Laboratories (Bar Harbor, ME). Since our previous study demonstrated an early recovery of the CBF in female mice after BCAS [36], in the present study, evaluation of ZT-1a treatment on CBF recovery was not feasible in female mice, and all experiments were conducted using only male mice. To mimic clinical VCID, we employed a metal microcoil BCAS model in C57BL/6J mice (10–14 weeks old, weighing 20–30 g). Mice were anesthetized with 5% isoflurane vaporized in 100% O_2_ for induction, maintained at 1.5% isoflurane during surgery, and placed in the supine position. Through a midline skin incision of the neck, both common carotid arteries (CCAs) were carefully isolated from the vagus nerves and tissues. Arteries were gently lifted by a 6−0 suture. A metal microcoil of 0.18-mm inner diameter was placed by twine around the right CCA just below the bifurcation to produce carotid stenosis. Similarly, another 0.16-mm diameter microcoil was placed around the left CCA. Sham control underwent identical surgical procedures without microcoil placement. The incision was sutured, and bupivacaine (100 µl 0.25%, infiltrated topically) and ketoprofen (1 mg/kg, intraperitoneal, i.p.) injections were administered to alleviate the pain induced by surgery. The animal was left in a cage and closely monitored for recovery from anaesthesia. Respiratory function was monitored, and body temperature was maintained at 36.5 ± 0.5 °C throughout the surgery using a heating pad, pulse oximetry, and a CO_2_ monitoring system (PhysioSuite, Kent Scientific). After recovery, animals were returned to their cages with free access to food and water.

### CBF measurement

**Fig. 1:**
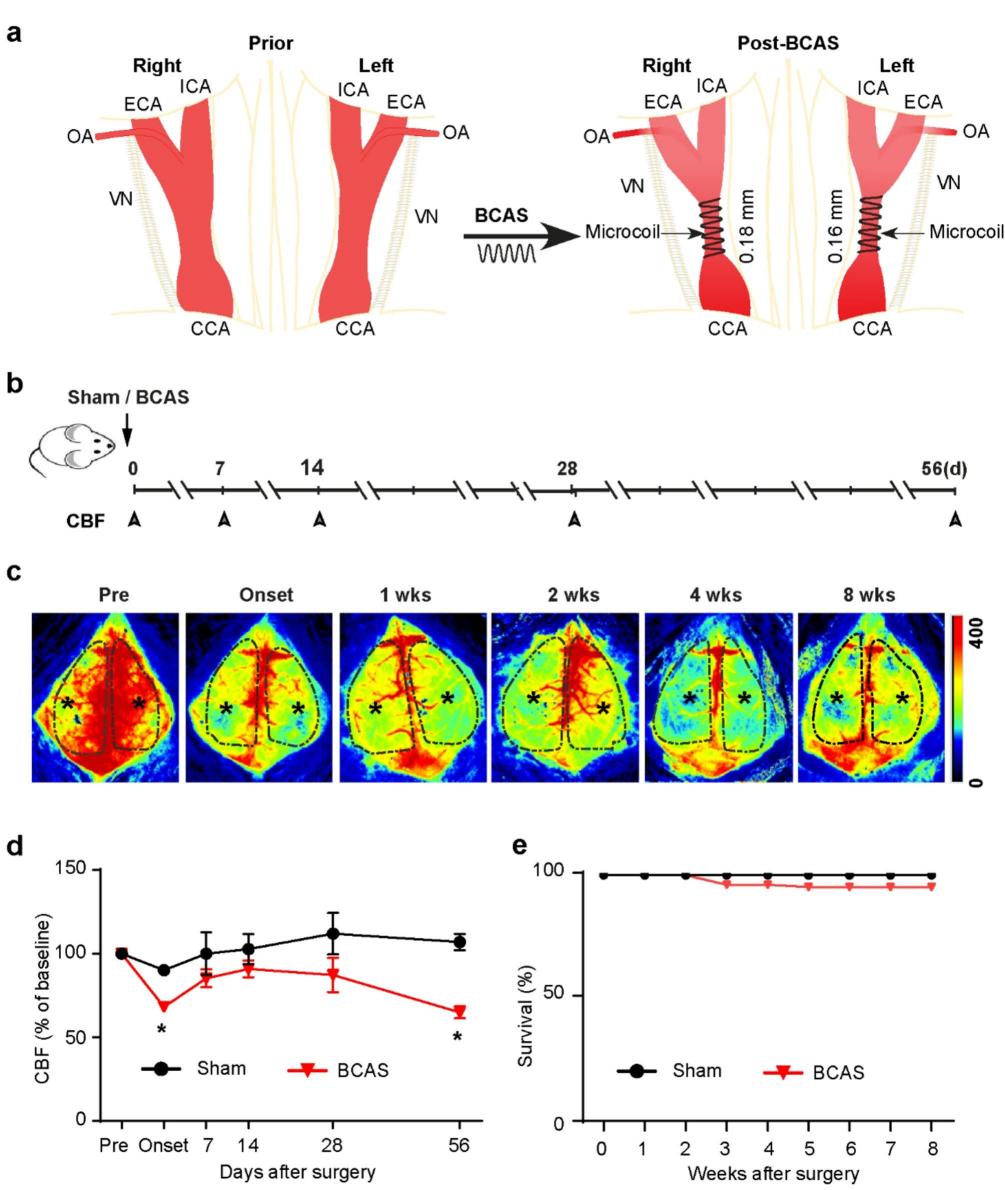
Bilateral carotid artery stenosis (BCAS) leads to a biphasic reduction in CBF in mice. **a** Schematic illustration of the metal microcoil bilateral carotid artery stenosis (BCAS) model. CCA, common carotid artery; d, days; ECA, external carotid artery; ICA, internal carotid artery; OA, occipital artery; VN, vagus nerve. **b** Experimental protocol and measured outcomes. Mice underwent Sham or BCAS procedure. Arrowheads below the timeline show the time point at which CBF was measured. CBF- cerebral blood flow. **c** Representative images of CBF captured via a two-dimensional laser speckle imaging system before, at the onset (10 min after surgery), 1 week, 2, 4, and 8 weeks after Sham or BCAS surgery. Black stars indicate brain regions with reduced CBF. Black dotted area: region of interest (ROI) used to quantify CBF. **d** Quantification of average CBF from the two hemispheres of the black dotted area in c. Data are presented as mean ± SEM; Two-way ANOVA, Tukey’s post-hoc test; *n* = 5–9, *p < 0.05, Sham vs. BCAS. **e** Percent survival over 0–8 weeks following BCAS surgery. *n* = 42–104

To measure CBF in mice, a two-dimensional laser speckle contrast analysis system (PeriCam PSI High Resolution with PIMSoft, Perimed, Sweden) was used as previously described [7]. A midline incision was made on the scalp, and the exposed skull surface was cleaned with sterile normal saline after mice were anesthetized with isoflurane (5% isoflurane vaporized in 100% O_2_ for induction and 1.5% for maintenance). Raw speckle images of the regions of interest (ROIs) covering the parietal lobe of each hemisphere were captured using a camera positioned 10 cm above the skull. Laser speckle images were captured at baseline, 10 min, 7-, 14-, 28-, and 56 days post-surgery (Fig. 1b). To minimize the potential confounding effects of isoflurane [45], we maintained the administration time and concentration of isoflurane consistent for each animal (i.e., 5% isoflurane for induction and 1.5% in 100% O_2_ for maintenance throughout ~ 20-min surgical time). The body temperature was also maintained at 36.5 ± 0.5 °C during surgery with the use of a rodent heating pad and an anal thermometer (PhysioSuite, Kent Scientific).

### Administration of SPAK inhibitor ZT-1a

BCAS mice were randomly grouped to receive either Vehicle (Veh) DMSO (2 ml/kg) or ZT-1a (5 mg/kg/day, every three days) via i.p. injection from days 28 to 56 post-BCAS surgery (Fig. 4a). The ZT-1a dose and treatment frequency are based on our previous findings that a 5 mg/kg dose for day 1 remained effective until 7 days post-stroke [82], and every 3 days, treatment showed efficacy up to 35 days post-BCAS [9]. Sham mice (which underwent identical surgical procedures without microcoil placement) received no treatment.

### Morris water maze (MWM) test

The MWM test was performed in a blind manner on days 49–55 after BCAS surgery to measure learning and memory function in mice. The MWM paradigm is an open-field procedure in which mice learn to find a hidden platform to escape from a pool of water. A circular tank (120 cm diameter) was filled with water at 22 °C (± 1 °C) to a depth of 31 cm. A round (10 cm diameter) Plexiglass platform submerged 0.5 cm below water level was left in the exact location (31 cm from the edge) for the duration of each trial. The learning trials were conducted over 7 days, with three daily trials. For each of the three trials, mice were placed in the pool facing the wall at one of three starting locations (northeast, southeast, southwest) and allowed to swim for a maximum of 90 s. If a mouse found the platform, it was allowed to remain on it for 30 s. Mice not finding the platform within the 90 s windows were placed on the platform and allowed to stay there for 30 s. A probe trial was conducted on the 8th day of testing without the hidden platform to further assess spatial memory function. Each trial was videotaped via a ceiling-mounted video camera, and latency and swim speed were calculated. After each session, the mouse was placed in a staging cage with a clean, dry towel. To allow the mouse to thermoregulate, the cage was warmed with a heating bulb. Mice were identified, and those who exhibited abnormal behavior, including corkscrew swimming or floating, were excluded from the experiment.

### Immunofluorescence (IF) staining

IF staining was performed as described before [37]. Mice were deeply anesthetized with 4% isoflurane and transcardially perfused with 0.1 M PBS (pH 7.4), followed by ice-cold 4% PFA in 0.1 M PBS, as described before [9]. Brains were cryoprotected with 30% sucrose after an overnight post-fixation in 4% PFA. Coronal Section (20 μm, at 1.18 mm anterior to bregma) were selected and processed for IF staining. The sections were incubated with blocking solution (10% normal goat serum, 3% bovine serum albumin, and 0.15% Triton X-100 in PBS) for 1 h at room temperature. They were then incubated with primary antibodies against C3d (goat), MBP (rabbit), SMI 32 (rabbit), tNKCC1 (T4, mouse), pNKCC1 (rabbit), GFAP (mouse), Iba1 (rabbit), NeuN (mouse), APC (mouse), NG2 (rabbit), LCN2 (rat), MMP-2 (rabbit), MMP-9 (rabbit), VEGFA (rabbit), ZO-1 (rabbit), GLUT1 (mouse), Albumin (rabbit), AQ4 (rabbit), TL488, and TL546 in blocking solution at 4 °C overnight. Dilution of the antibodies and vendor information were included in Table S1 of the Additional File. To visualize the BBB integrity protein Claudin-5, sections were incubated with 0.5 mg/ml Actinase E enzyme in 0.15% Triton X-100 in PBS at 37 °C for 10 min. Then permeabilized in 0.5% Tx-100 in PBS for 10 min at RT, followed by incubation with primary antibodies against Claudin-5. After washing in TBS three times for 10 min, the sections including Claudin-5 were incubated with goat anti-mouse Alexa 488-conjugated IgG and goat anti-rabbit Alexa 546-conjugated IgG in the blocking solution for 1 h. For negative controls, brain sections were stained with secondary antibodies only. After washing three times, nuclei were stained with DAPI for 20 min at 37 °C. Sections were mounted on slides using a Vectashield mounting medium (Vector Laboratories). Fluorescent images were captured under a 40× lens with 2× magnification using a Nikon A1R confocal or Olympus 1000 inverted confocal laser-scanning microscope. Identical digital imaging acquisition parameters were used, and images were obtained and analyzed in a blinded manner throughout the study. For each mouse, a minimum of 3 brain slices were stained, and images were captured from 3 non-overlapping fields per slice. The mean values were calculated for each mouse and used for statistical analysis. Astrocytic endfeet coverage was measured from immunoassayed images of AQP4 (an astrocytic endfeet marker) and tomato lectin (TL, a blood vessel marker) using ImageJ software. Briefly, the color thresholds of the raw TIFF images, captured via a confocal microscope, were adjusted to obtain the color hue of the covered area of the vessels. Then the desired color was selected to measure the total area covered with that color. The astrocytic endfeet coverage around the blood vessel was calculated by the ratio of the yellow and reddish-yellow hues (the merged area of the astrocytic endfeet marker AQP4 and blood vessel marker TL) to the total green hues (the total area of the blood vessel expressed as the total green hues of TL). At least five blood vessels from each mouse sample were used for quantification and analysis.

### Western blotting

Mice brain homogenates were prepared as previously described [7]. The mice were deeply anesthetized with 4% isoflurane, transcardially perfused with saline, and subsequently decapitated. The brain was dissected on ice, and the tissues were collected in 7 volumes of ice-cold homogenization buffer, comprising Tissue Protein Extraction Reagent (T-PER^TM^, Thermo Scientific) and a protease and phosphatase inhibitor cocktail (Pierce). The tissues were homogenized using a tissue grinder (Kontes, Vineland, NJ, USA) for 10 strokes in the homogenization buffer, followed by centrifugation at 1000 g at 4 °C for 10 min. Protein concentration was determined by the bicinchoninic acid (BCA) assay. Protein samples (10 µg) were boiled in sample buffer (Thermo Scientific, Rockford, IL, USA) at 100 °C for 5 min, then resolved via 10% sodium dodecyl sulfate-polyacrylamide gel electrophoresis (SDS-PAGE) and transferred onto a polyvinylidene difluoride (PVDF) membrane. The membrane was incubated overnight at 4 °C with appropriate primary antibodies anti-synaptophysin (SYP); anti-postsynaptic density protein-95 (PSD-95). Subsequently, the membrane was washed with TBST (Tris-buffered saline, with 0.05% Tween-20) followed by incubation for 1 h at room temperature with horseradish peroxidase conjugated secondary antibodies (anti-rabbit or anti-mouse, dilution 1:2000). Protein bands were visualized using enhanced chemiluminescence (ECL) reagents. Densities of the bands were measured using ImageJ software. GAPDH was used as a loading control for total protein normalization.

### Statistical analysis

Animals were randomly allocated to different studies and surgical procedures. All data analyses were conducted by investigators blinded to experimental conditions. The number of animals studied was 80% powered to detect 25% changes with α (two-sided) = 0.05. All mice were included in the study, except for seven, which died 1–56 days after BCAS surgery. Data were expressed as mean ± SEM. All data were evaluated for normal distribution using the D’Agostino and Pearson normality test and the Shapiro–Wilk normality test. Student’s t-test, or one-way or two-way ANOVA, followed by Tukey’s post hoc test for multiple comparisons (GraphPad Prism 6.0, San Diego, CA, USA), were used to determine statistical differences between groups. The two-way repeated-measures ANOVA followed by Tukey’s post-hoc was used to evaluate the escape latency times in the MWM test. A probability value less than 0.05 was considered statistically significant.

## Results

### The BCAS mouse model exhibited a biphasic reduction in CBF

Most patients with intracranial atherosclerotic stenosis clinically exhibit permanent narrowing without complete occlusion [21], often showing unilateral or asymmetric stenosis [12, 62]. To mimic clinical atherosclerotic stenosis with sustained hypoperfusion of the CCA, we utilized an asymmetric BCAS model (Fig. 1a) by twining CCAs with 0.18 mm (right CCA) and 0.16 mm (left CCA) inner diameter microcoils in adult C57BL/6J mice. Laser speckle imaging of CBF shows sustained cerebral hypoperfusion over 0–8 weeks post-BCAS (Fig. 1c). Compared to Sham, the asymmetric BCAS procedure in mice led to a sudden drop in CBF (~ 31%, *p < 0.05, Fig. 1d) in the first 10 min of BCAS and then partially recovered over 1–4 weeks.

Interestingly, BCAS mice exhibited a second episode of sustained CBF reduction at 4–8 weeks post-BCAS (~ 35%, *p < 0.05, Fig. 1d). The asymmetric BCAS model exhibited a mortality rate of ~ 5% within 8 weeks post-surgery (Fig. 1e). Although our asymmetric BCAS model resulted in identical lesions in both hemispheres as assessed by MBP loss in corpus callosum (CC, data not shown), we reported the average data collected from both hemispheres in this study.

### Reactive astrogliosis and microglial activation coincided with increased total and phosphorylated NKCC1 levels during the early stage of VCID

**Fig. 2:**
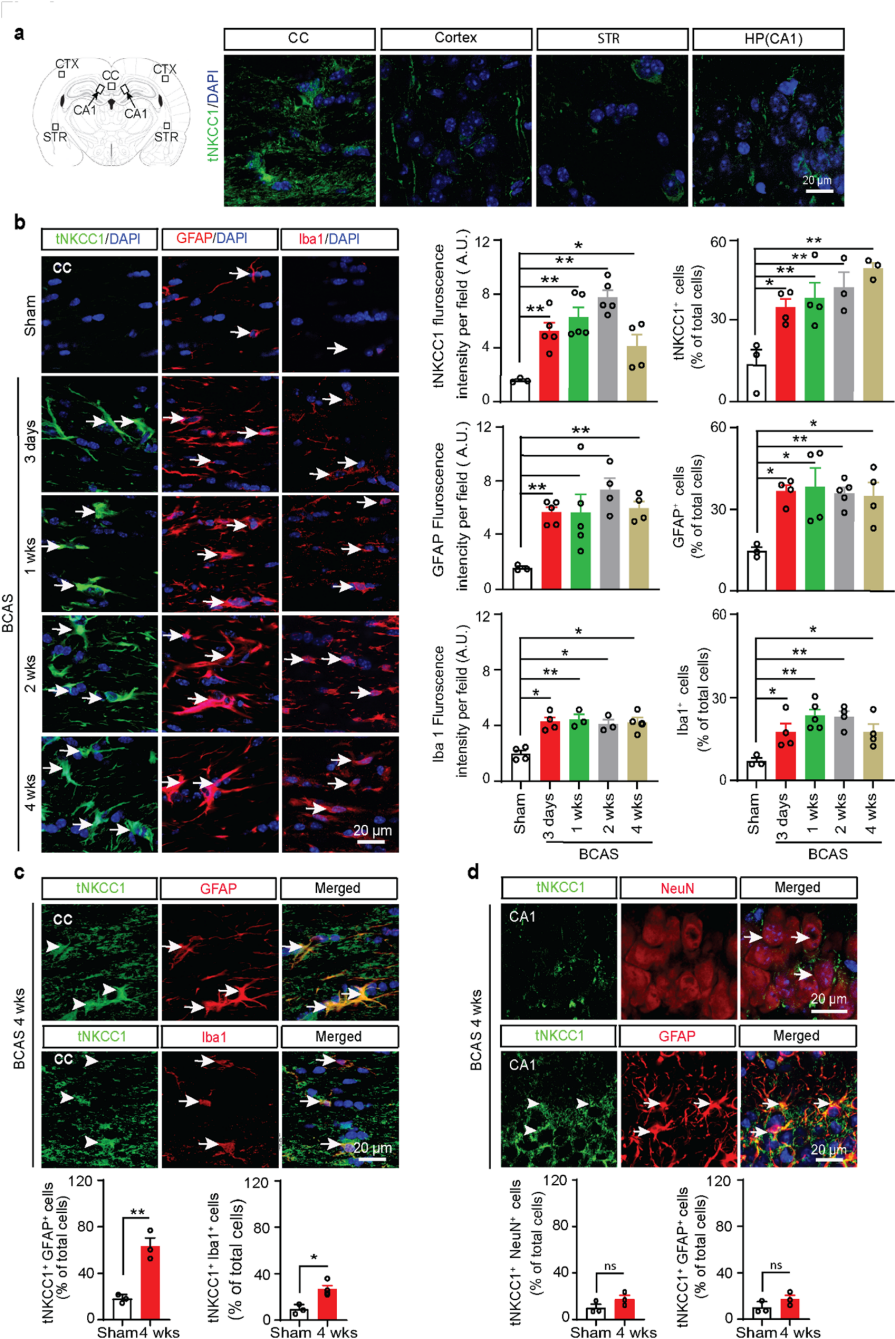
BCAS induces NKCC1 protein upregulation in GFAP^+^ astrocytes and Iba1^+^ microglia/macrophages in white matter tracts. **a** The brain section (left panel) illustrates sample collection from the CTX, CC, striatum (STR), and hippocampal CA1 subfield (CA1). A robust increase in tNKCC1 expression (right panel) was observed in the CC compared to CTX, STR, and hippocampal CA1 areas at 4 weeks post-BCAS. **b** Representative images of the time-dependent expression of tNKCC1, GFAP, and Iba1 at 3 days, 1 week, 2 weeks, and 4 weeks after BCAS. Compared to Sham-Ctrl, tNKCC1^+^, GFAP^+^, and Iba1^+^ cells significantly increased at 3 days in the CC of the BCAS mice brain and remained elevated until 4 weeks after BCAS. Data are mean ± SEM; one-way ANOVA, Tukey’s post-hoc test; *n* = 3–5; *p < 0.05, **p < 0.01. **c**-**d** The increase in tNKCC1 protein expression (arrowhead) colocalized with both GFAP^+^ astrocytes (arrow) and Iba1^+^ microglia/macrophages (arrow) in the CC and in GFAP^+^ astrocytes (arrow) in the hippocampal CA1 regions but not in the NeuN^+^ neurons at 4 weeks after BCAS. Data are mean ± SEM; one-way ANOVA, Tukey’s post-hoc test; *n* = 3–4; *p < 0.05, **p < 0.01, ns = not significant

**Fig. 3:**
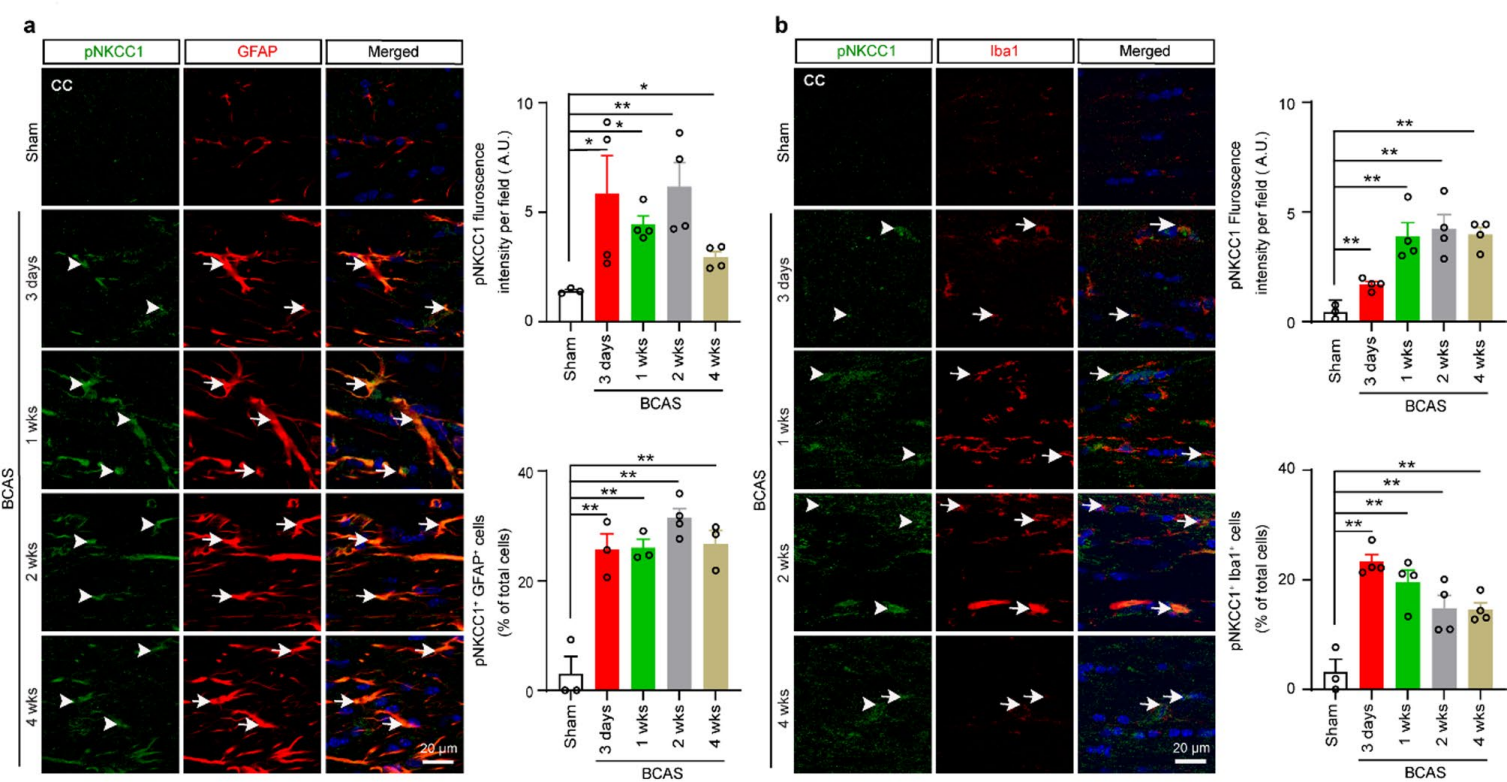
BCAS triggers early phosphorylation of NKCC1 in astrocytes and microglia/macrophages. Time-dependent expression of pNKCC1 in GFAP^+^ astrocytes (**a**) and Iba1^+^ microglia/macrophage (**b**) at 3 days, 1 week, 2, and 4 weeks after BCAS. Compared to Sham-Ctrl, both pNKCC1^+^ GFAP^+^ cells and pNKCC1^+^ Iba1^+^ cells showed a significant increase in the CC of the BCAS mice brain 3 days after BCAS and remained elevated until 4 weeks. Arrowheads indicate pNKCC1 expressions **a**-**b**. Arrows indicate GFAP (**a**) and Iba1 expressions (**b**), respectively. Data are mean ± SEM; one-way ANOVA, Tukey’s post-hoc test; *n* = 3–4; *p < 0.05, **p < 0.01

At first, we assessed the expression of total NKCC1 (tNKCC1) protein in different regions of the brain, including the cortex (CTX), striatum (STR), hippocampal CA1, and CC (Fig. 2a), 4 weeks after BCAS surgery. Among these regions, white matter CC exhibited the most robust expression of tNKCC1 (Fig. 2a). Therefore, we focused on examining the relation between tNKCC1 activity and white matter-reactive astrogliosis, along with white matter lesions (WML) in the CC and external capsule (EC) tracts. To assess the temporal expression of tNKCC1, Iba1 (a marker for microglia activation) and GFAP (a marker for reactive astrocytes) in white matter, we performed IF analyses of mouse brain tissue samples collected at 3-days, 1-, 2-, and 4-weeks after BCAS. Compared to Sham, tNKCC1 fluorescence intensity increased 5.8 folds 3 days after BCAS (Fig. 2b, **p < 0.01), further elevated to 6.8 folds at 2 weeks (**p < 0.01), and then remained elevated until 4 weeks after BCAS. Similarly, GFAP and Iba1 fluorescence intensities showed significant increases 3 days after BCAS and remained higher until 4 weeks after BCAS (Fig. 2b). These data show that both reactive astrogliosis and microglial activation occurred concurrently with the upregulation of NKCC1 expression at an early stage after BCAS, suggesting a potential link between tNKCC1 and the inflammatory processes. To identify the responsible cell types for the elevated tNKCC1 expression, we checked tNKCC1 expression in Iba1^+^ microglia/macrophages in CC, GFAP^+^ astrocytes in both CC and hippocampal CA1 region, and NeuN^+^ neurons in hippocampal CA1 region. Most of the GFAP^+^ astrocytes were positive for increased tNKCC1 expression (~ 63% of total cells, Fig. 2c), whereas a few Iba1^+^ microglia/macrophages were colocalized with tNKCC1 immunoreactivity (~ 36% of total cells, Fig. 2c). However, increased tNKCC1 expression was not colocalized in NeuN^+^ neurons in the hippocampal CA1 region, rather in GFAP^+^ astrocytes (Fig. 2d). We then analysed phospho-NKCC1 (pNKCC1) expression and found that pNKCC1 also followed similar pattern as tNKCC1 and profoundly colocalized with GFAP^+^ astrocytes, modestly in Iba1^+^ microglia/macrophages (Fig. 3a, b). Collectively, these results suggest that hypoperfusion triggers microglial activation and reactive astrogliosis through increased NKCC1 activity, predominantly in the white matter tracts, and, to a lesser extent, in the hippocampal CA1 region.

### ZT-1a inhibited NKCC1 expression and phosphorylation, microglial activation, reactive astrogliosis, and cytotoxic LCN2 expression in BCAS mice

**Fig. 4:**
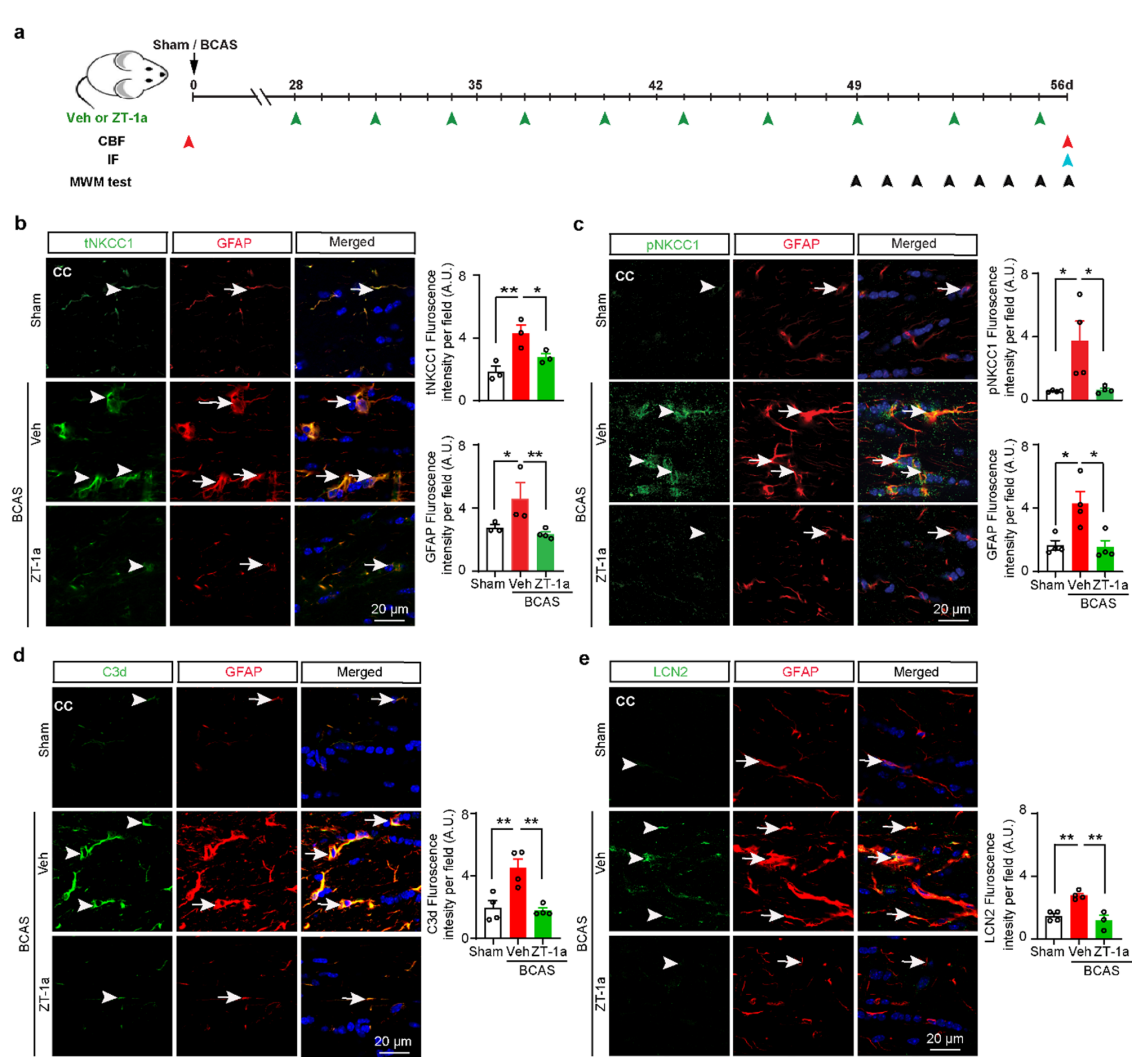
ZT-1a inhibits NKCC1 phosphorylation and downregulates NKCC1, C3d, and LCN2 expression in white matter astrocytes. **a** The graphical presentation of the experimental protocol. Mice went through either Sham or BCAS surgery. BCAS mice were administered either Veh (100% DMSO, 2 ml/kg) or SPAK inhibitor ZT-1a (5 mg/kg) via intraperitoneal injection (i.p.) for 28–56 days (every 3 days) after BCAS, green arrowheads indicate the time points of Veh or drug treatment. Red, light blue, and black arrowheads indicate the respective time points for cerebral blood flow (CBF) measurement, immunofluorescence (IF) staining, and Morris water maze (MWM) test. (b-c) ZT-1a-treated mice exhibit reduced expression of tNKCC1 and pNKCC1 in GFAP^+^ reactive astrocytes in the CC compared to Veh-treated mice at 8 weeks after BCAS surgery. Arrowheads indicate tNKCC1 (**b**) and pNKCC1 (**c**) expressions, respectively. Arrows indicate GFAP expression (b-c). Data are mean ± SEM; one-way ANOVA, Tukey’s post hoc test; *n* = 3–4; *p < 0.05, **p < 0.01. Compared to Veh-treated mice, ZT-1a-treated mice exhibit decreased C3d (**d**) and LCN2 (**e**) expressions in GFAP^+^ reactive astrocytes in CC at 8 weeks post-BCAS. Arrowheads indicate C3d (d) and LCN2 (e) expressions, respectively. Arrows indicate GFAP expression (d-e). Data are mean ± SEM; one-way ANOVA, Tukey’s post hoc test; *n* = 3–4; *p < 0.05, **p < 0.01

Next, we examined whether treatment with SPAK inhibitor ZT-1a decreased BCAS-induced expression of tNKCC1, pNKCC1, and attenuated microglial activation and reactive astrogliosis in the white matter tracts. IF analysis showed an increased tNKCC1 fluorescence intensity along with both GFAP and Iba1 intensity in the CC region of the Veh-treated group, relative to the Sham group (**p < 0.01, Fig. 4b and Additional File: Fig. S2c). As expected, ZT-1a treatment markedly reduced BCAS-induced phosphorylation of NKCC1 (pNKCC1) and expression of tNKCC1, GFAP and Iba1 in the CC region of the white matter (Fig. 4c and Additional File: Fig. S2d). Collectively, these results suggest that ZT-1a treatment reduced BCAS-induced reactive astrogliosis and microglial activation by inhibiting the activity of the SPAK-NKCC1 complex.

Proinflammatory reactive astrogliosis is the hallmark of different dementing disorders, including VCID. Recent studies showed that CCH increases the number of cytotoxic reactive astrocytes [44]. Therefore, we also examined whether ZT-1a treatment attenuates the transformation of homeostatic astrocytes into cytotoxic reactive astrocytes by IF analysis using a specific antibody against C3d (a cytotoxic reactive astrocyte marker). Veh-treated BCAS mice exhibited increased C3d expression compared to Sham mice (**p < 0.01), while ZT-1a treatment attenuated C3d expression compared to Veh-treated BCAS mice (**p < 0.01, Fig. 4d). These results suggest that ZT-1a blocks the transformation of homeostatic astrocytes into cytotoxic reactive astrocytes in VCID. Lipocalin-2 (LCN2), a member of the highly heterogeneous secretory protein family of lipocalins, is a potential diagnostic biomarker of several neurological diseases, including AD and vascular dementia [14, 44]. Previous studies revealed that under inflammatory conditions, reactive astrocytes secrete LCN2, which contributes to the death of several brain cells, including neurons [10, 40]. Therefore, we examined whether BCAS induces LCN2 expression in reactive astrocytes using anti-GFAP and anti-LCN2 antibodies. We also observed LCN2 expression exclusively colocalized with GFAP^+^ astrocytes but not with Iba1^+^ microglia/macrophages after 4 weeks post BCAS (data not shown). Veh-treated BCAS mice showed an elevated level of LCN2 in GFAP^+^ reactive astrocytes compared to Sham in the CC of the white matter tracts (**p < 0.01, Fig. 4e) at 8 weeks post-BCAS. Interestingly, 4–8 weeks of ZT-1a treatment in BCAS mice significantly decreased the level of LCN2 expression compared to Veh-treated BCAS mice (**p < 0.01). These data suggest that ZT-1a attenuates reactive astrogliosis and astrocytic LCN2-mediated cytotoxicity to brain cells in the white matter and HP in BCAS mice.

### ZT-1a protected BBB integrity and restored CBF in BCAS mice

**Fig. 5:**
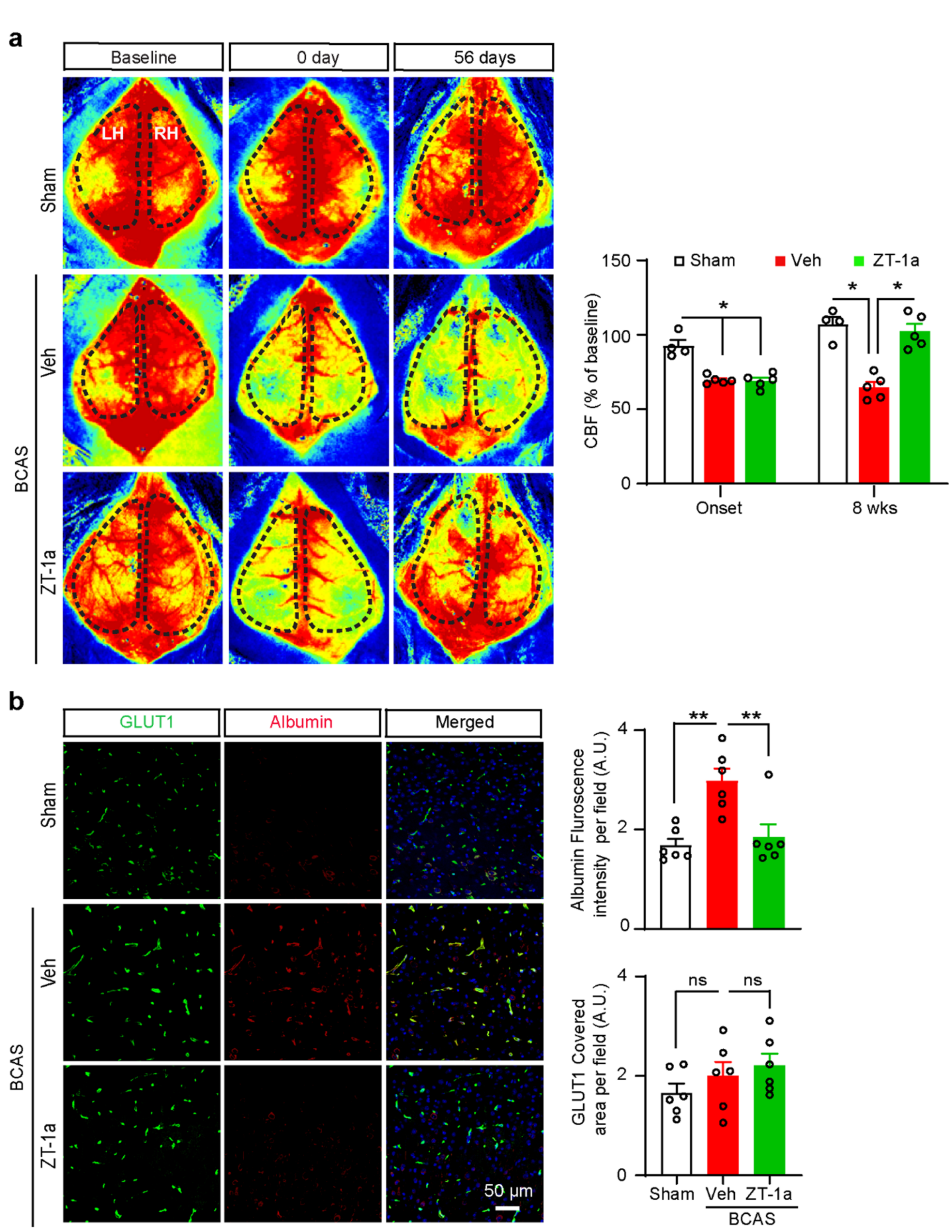
ZT-1a restores CBF and prevents BBB leakage in the mouse brain 8 weeks post-BCAS. **a** Based on the timeline presented in Fig. 1b, days 0 and 56 were selected as timepoints to assess the effect of ZT-1a in a separate experiment with different groups of mice. Representative images of the CBF in the brains of Sham, Veh-, and ZT-1a-treated mice during the 0–8 weeks after BCAS. Baseline (before surgery), 0 d (10 min after surgery), and 56 d (8 weeks after surgery). Black dotted area: region of interest (ROI) used to quantify CBF. Bar graphs show increased CBF in ZT-1a-treated mice brains compared to Veh-treated mice at 8 weeks post-BCAS. Data are mean ± SEM; one-way ANOVA, Tukey’s post hoc test; *n* = 4–5; *p < 0.05. LH, left hemisphere; RH, right hemisphere **b** Representative immunofluorescence images of GLUT1 and albumin in the cortex (CTX) of Sham, Veh- and ZT-1a-treated mice brain at 8 weeks of BCAS. Compared to Veh-treated mice, ZT-1a treatment exhibited reduced albumin fluorescence intensity in the brain. Data are mean ± SEM; one-way ANOVA; Tukey’s post hoc test; *n* = 4–5, *p < 0.05, ns = not significant

Next, we investigated whether ZT-1a treatment preserves BBB integrity and restores CBF in mice at the advanced stage of VCID. Laser speckle imaging showed an approximate 31% reduction in CBF at the onset (10 min after BCAS) of the BCAS procedure in both Veh and ZT-1a assigned groups compared to the Sham group (Fig. 5a). As shown in Fig. 4a, BCAS mice received either Veh or ZT-1a treatment regimens during days 28–56 post-BCAS. At the end of the treatment (at 8 weeks post-BCAS), Veh-treated mouse brains exhibited a significant reduction in CBF compared to Sham brains (*p < 0.05, Fig. 5a). In contrast, the ZT-1a-treated mice showed an increased CBF in comparison to Veh-treated mice (*p < 0.05). These results led us to test BBB integrity and astrocytic endfeet as possible explanations for the observed changes in CBF.

CBF regulation depends on the integrity of the BBB, and the presence of extravasated albumin in the brain parenchyma indicates BBB disruption, making it a reliable marker for assessing BBB integrity [47]. BBB leakage is also one of the key features following CCH. Therefore, we evaluated whether changes in BBB integrity are involved in the CBF deregulation following BCAS. We determined BBB leakage in the subcortical area of the mouse brain at 8 weeks post-BCAS through IF staining with anti-albumin and anti-GLUT1 (a blood vessel marker) antibodies. A significant increase in albumin fluorescence intensity was observed in Veh-treated mouse brains compared to Sham brains (*p < 0.05, Fig. 5b), while a reduced albumin fluorescence intensity was observed in ZT-1a-treated mouse brains compared to Veh-treated mouse brains. There was no significant change in vessel areas, as the vessel (GLUT1^+^) areas were similar in all groups (Fig. 5b). These findings indicate that selective inhibition of SPAK-NKCC1 activity by ZT-1a prevents BBB leakage in the mouse brain following BCAS.

**Fig. 6:**
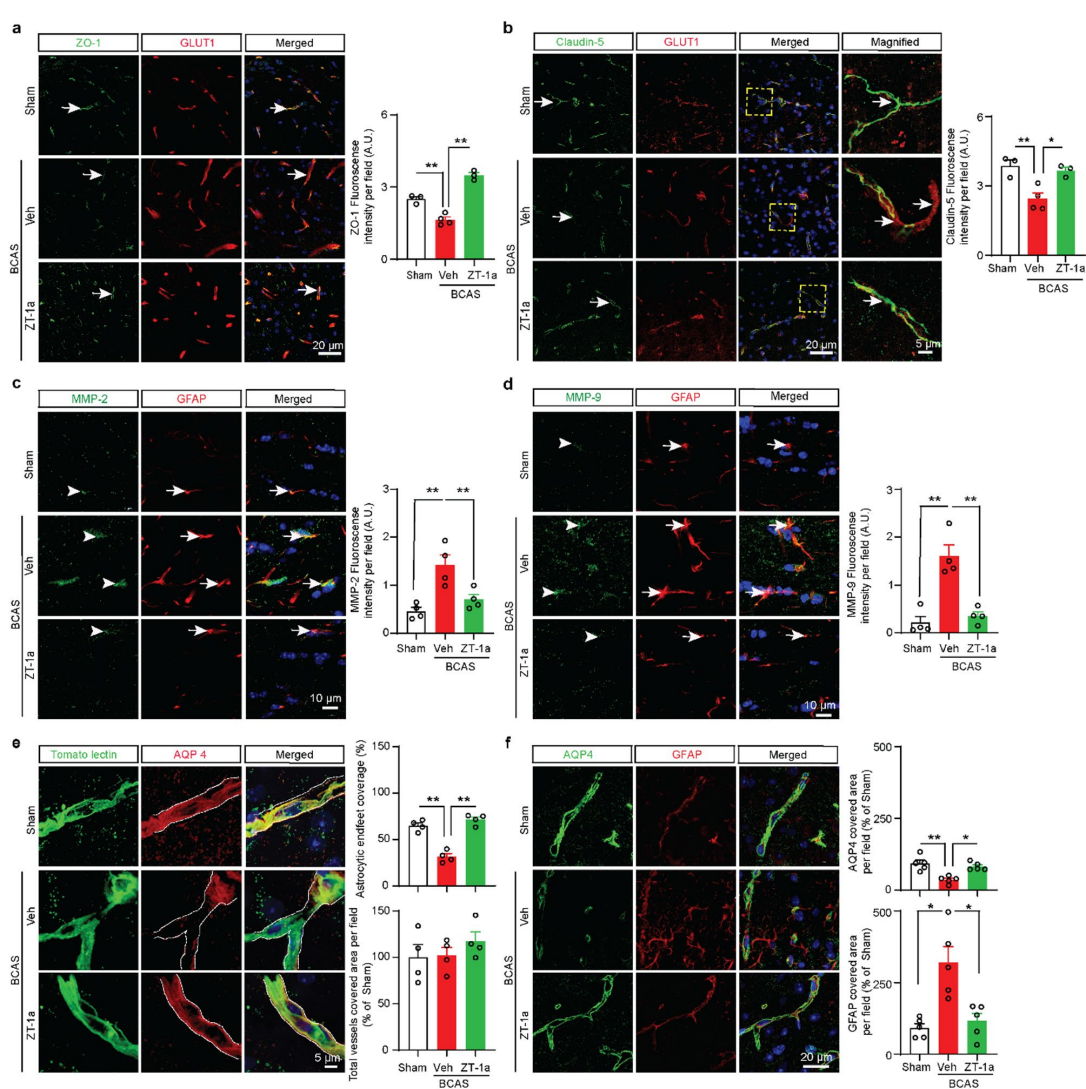
ZT-1a preserves BBB integrity, reduces MMP-2/9 expression, and improves astrocytic endfeet coverage 8 weeks post-BCAS. **a** Immunofluorescence analysis of Zonula 1 (ZO-1) and the vessel marker GLUT1 in the cortex of Sham, Veh-, and ZT-1a-treated mice brain at 8 weeks post-BCAS. Arrows indicate ZO-1 expression. Quantitative analysis of ZO1 fluorescence intensity. Data are mean ± SEM; one-way ANOVA; Tukey’s post hoc test; *n* = 3–4, **p < 0.01. **b** Representative images of Claudin-5 and GLUT1 in the cortex (CTX) of Sham, Veh-, and ZT-1a-treated mice brain at 8 weeks of BCAS. Arrows indicate Caludin-5 expression. Data are mean ± SEM; one-way ANOVA; Tukey’s post hoc test; *n* = 3–4, *p < 0.05, **p < 0.01. **c**-**d** Compared to Veh-treated BCAS mice, ZT-1a-treated mice exhibited significantly reduced MMP-2 and MMP-9 expressions in GFAP^+^ astrocytes in the CC at 8 weeks post-BCAS. Data are mean ± SEM; one-way ANOVA; Tukey’s post hoc test; *n* = 3–4, **p < 0.01. Arrowheads indicate expression of MMP-2 (c) and MMP-9 (d), respectively. Arrows indicate GFAP expression (**c**-**d**). **e** Immunofluorescence analysis of tomato lectin and AQP 4 in the cortex (CTX) after 8 weeks of BCAS. The white dotted area: Region of interest (ROI) presents the quantified region for tomato lectin and AQP 4. The coverage of astrocytic endfeet around the vessels was determined by dividing the total area of the vessels (tomato lectin) by the total merging area of the astrocytic endfeet (AQP4) and the vessels (tomato lectin). The bar graph shows increased astrocytic coverage in ZT-1a-treated mice compared to Veh-treated mice. At least 5 vessels were analyzed for quantification. Data are mean ± SEM; one-way ANOVA; Tukey’s post hoc test; *n* = 3–4, **p < 0.01. **f** The representative images of the GFAP and AQP4 immunostained cortex (CTX) samples of Sham, Veh-, and ZT-1a-treated mice brain at 8 weeks after BCAS surgery. The covering area of astrocytic endfeet (AQP4) and astrocytes (GFAP) were measured using ImageJ software. Data are mean ± SEM; one-way ANOVA; Tukey’s post hoc test; *n* = 6–10, *p < 0.05, **p < 0.01

Next, we examined the expression of TJ proteins ZO-1 and Cluadin-5. As shown in Fig. 6a, b, the expression of ZO-1 and Cludin-5 were nicely colocalized with GLUT1^+^ vessel (arrow indicates colocalization). Compared to Sham mice, Veh-treated BCAS mice exhibited a significantly reduced level of ZO-1 and Claudin-5, suggesting a compromise in BBB structural integrity in BCAS mice. Importantly, ZT-1a-treated BCAS mice showed a significantly increased level of both ZO-1 (**p < 0.01) and Cluadin-5 (*p < 0.05) compared to Veh-treated BCAS mice. Collectively, these findings strongly suggest that ZT-1a treatment mitigates BCAS-induced BBB leakage by preserving TJ components.

### ZT-1a attenuated BCAS-induced astrocytic MMP-2 and MMP-9 expression and preserved astrocytic endfeet around microvessels in mice

We found that ZT-1a treatment restores CBF 8 weeks after BCAS, although the underlying mechanism remains unclear. We investigated whether ZT-1a has any effect on VEGF expression (a marker of angiogenesis) [54], formation of new vessels (measured by GLUT1 expression), and expression of matrix metalloproteinases (MMPs). We found that ZT-1a treatment did not affect angiogenesis, as indicated by unchanged VEGF expression and vessel area in both Veh- and ZT-1a-treated mouse brains (Additional File: Fig. S4b). MMPs, particularly MMP-2 and MMP-9, are known to play a crucial role in BBB disruption during ischemic stroke [69]. To assess MMP-2 and MMP-9 levels, we immunostained brain samples from Sham, Veh-, and ZT-1a-treated mice using anti-MMP-2, anti-MMP-9, and anti-GFAP antibodies. Our results showed a significant increase in both MMP-2 (**p < 0.01) and MMP-9 (**p < 0.01) intensity in Veh-treated BCAS mice (Fig. 6c, d). However, ZT-1a-treated BCAS mice exhibited a significant reduction in MMP-2 (**p < 0.01) and MMP-9 (**p < 0.01) compared to Veh-treated BCAS mice. Interestingly, both MMP-2 and MMP-9 colocalized with GFAP⁺ astrocytes (Fig. 6c, d). These findings suggest that astrocytic MMP-2 and MMP-9 contribute to BBB damage by degrading BBB-associated proteins, whereas ZT-1a mitigates BBB damage by attenuating astrocytic MMP-2/9 expression.

We also examined the endfeet and pericyte coverage around the microvessels in BCAS mouse brains because astrocytic endfeet, in combination with pericytes, play a crucial role in maintaining the BBB integrity and CBF [39, 77]. We did not find any significant changes in pericyte coverage (PDGFRβ) around vessels (marked by tomato lectin) between the Sham and Veh-treated BCAS groups (Additional File: Fig. S4c). We then evaluated astrocytic endfeet coverage around the microvessel using anti-AQP4 (an astrocytic endfeet marker) along with the vessel marker tomato lectin expression. The white dotted areas in Fig. 6e represent the calculated percentage of astrocyte endfeet coverage around microvessels. As shown in Fig. 6e, the BCAS group exhibited a significant reduction in astrocytic endfeet coverage compared to the Sham group (**p < 0.01), indicating the detachment of astrocytes from microvessels. However, a significantly increased percentage of astrocytic endfeet ensheathing microvessels was observed in the ZT-1a-treated group compared to the Veh-treated group (**p < 0.01), suggesting a restorative effect. To rule out the possibility that astrocytic endfeet loss in Veh-treated BCAS mice was not due to loss of astrocytes, we analyzed the AQP4^+^ endfeet coverage along with GFAP^+^ astrocyte coverage. Although we observed a significant reduction (**p < 0.01) in AQP4^+^ astrocytic endfeet, a GFAP^+^ astrocyte density/ROI was indeed higher in Veh-treated BCAS mice compared to Sham mice (Fig. 6f), supporting our hypothesis that CCH specifically contributes to the loss of astrocytic endfeet al.ong the vessels but not to the loss of total astrocytic coverage. Together, these findings suggest that astrocytic endfeet loss impairs BBB integrity and neurovascular coupling, leading to a reduction in CBF at delayed time points after BCAS, while ZT-1a treatment enhances CBF by mitigating these effects.

### ZT-1a treatment during the symptomatic stage of VCID reversed white matter demyelination and prevented delayed hippocampal neuronal loss

**Fig. 7:**
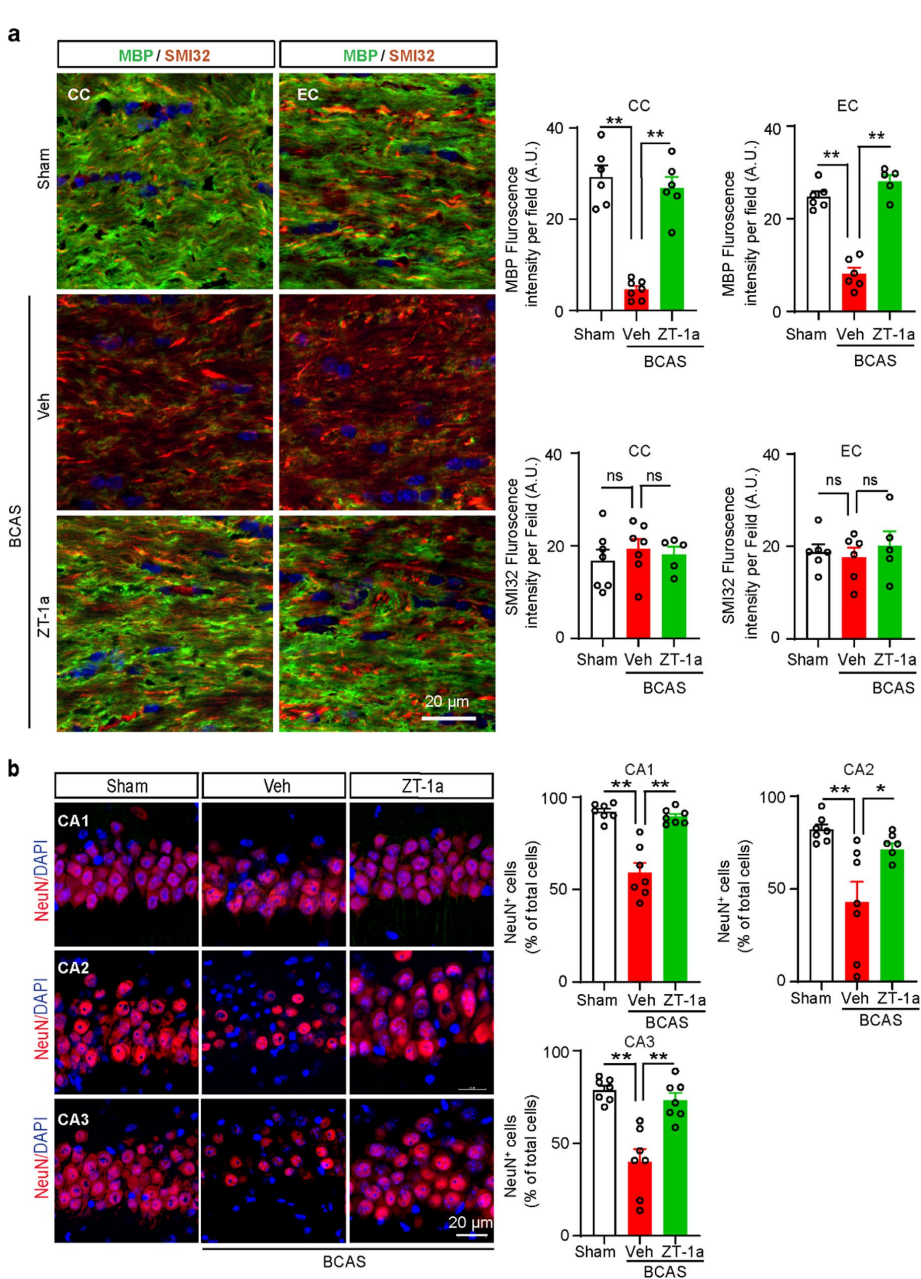
ZT-1a reverses BCAS-induced MBP loss and prevents hippocampal neuronal death 8 weeks post-BCAS. **a** Immunostaining analysis of myelin basic protein (MBP) and nonphosphorylated neurofilament heavy chain (SMI32) in CC and EC of the mice brain at 8 weeks post-BCAS. Compared to Veh-treated mice, ZT-1a treatment significantly prevents BCAS-induced MBP loss at 8 weeks after BCAS. Quantitative analysis of MBP and SMI32 immunofluorescence (right panels). Data are mean ± SEM, one-way ANOVA, Tukey’s post hoc test; *n* = 5–7, **p < 0.01, ns = non-significant. **b** ZT-1a-treated mice show an increase in NeuN^+^ cells in the hippocampus in comparison to Veh-treated mice at 8 weeks post-BCAS. Quantitative analysis. Data are mean ± SEM; one-way ANOVA; Tukey’s post hoc test; *n* = 6–7, **p < 0.01

Internal carotid artery stenosis-induced cerebral hypoperfusion can cause white matter lesions (WML) and cognitive impairment in humans [17, 56]. Using the BCAS animal model of VCID, several studies have shown that demyelination in the white matter tracts, especially in the CC, is associated with cognitive impairment, and mitigating this myelin loss improves cognitive function [42, 43]. Therefore, we assessed the demyelination and axonal degeneration induced by BCAS in the white matter after 8 weeks of BCAS. Double IF analysis of MBP (a myelin marker) and SMI32 (a marker for axonal degeneration) revealed a significant loss of MBP protein (p**<0.01) at 8 weeks after BCAS, with no detectable changes in SMI32 in both the CC and external capsule (EC) regions of the mouse brains (Fig. 7a). We then checked whether ZT-1a can rescue BCAS-induced demyelination in white matter. The massive loss of MBP protein in Veh-treated mice was significantly rescued in ZT-1a-treated mice (p**<0.01), suggesting that ZT-1a treatment replenishes BCAS-induced loss of myelin in the white matter tracts. Next, we examined whether the preserved myelin in the ZT-1a-treated mice was due to increased oligodendrogenesis and/or decreased death of oligodendrocyte (OL)/oligodendrocyte precursor (OPC) cells. To test this hypothesis, we evaluated the OPC, NG2^+^ counts and differentiated mature OL, APC^+^ cell counts in Sham, Veh-, and ZT-1a-treated mice from the same cohort of brains (Additional File: Fig. S5). Compared to Sham mice, the Veh-treated BCAS mice exhibited a significant loss of APC^+^ (**p < 0.01) mature OL counts, and NG2^+^ (**p < 0.01) OPC cell counts in both CC and EC tracts at 8 weeks post BCAS (Additional File: Fig. S5). Conversely, significantly higher counts of both APC^+^ (**p < 0.01) and NG2^+^ (*p < 0.05) cells in the CC and EC tracts were observed in the ZT-1a-treated BCAS group in comparison to the Veh-treated mice (Additional File: Fig. S5). These findings suggest that ZT-1a treatment not only enhanced the differentiation and maturation of OPC into mature OL but also prevented cell death of both OPC and OL. Along with demyelination, a few reports implicated hippocampal degeneration as a contributing factor to memory impairment in the mouse model of BCAS [50]. We then examined whether delayed neurodegeneration occurs at 8 weeks post-BCAS and evaluated the effect of ZT-1a on BCAS-induced neuronal loss in the hippocampal CA1, CA2, and CA3 regions. Compared to the Sham group, Veh-treated mice exhibited a modest but significant reduction in NeuN^+^ cell counts (*p < 0.05, Fig. 7b) in the CA1 region. In contrast, a more pronounced decrease in NeuN^+^ cell counts was observed in both the CA2 (**p < 0.01) and CA3 (**p < 0.01) regions of Veh-treated brains relative to Sham-operated mice (Fig. 7b). However, treatment with ZT-1a for 4–8 weeks post-BCAS significantly (**p < 0.01) rescued this delayed neuronal loss. We also examined neuronal loss in the CTX region following BCAS and found no significant differences in NeuN^+^ cell counts among the groups (data not shown). We then assessed BCAS-induced synaptic loss in CTX and HP regions by Western blotting using the pre- and post-synaptic protein markers SYP and PSD-95. We did not observe any significant changes in the level of both SYP and PSD-95 proteins in the CTX and HP regions (Additional File: Fig. S6), which are in consistent with previous reports [4, 70]. Taken together, these results suggest that BCAS-mediated white matter demyelination and hippocampal neuronal death, but not synaptic loss, are associated with cognitive impairment following BCAS.

### ZT-1a treatment during the symptomatic stage of VCID rescued cognitive impairment

**Fig. 8:**
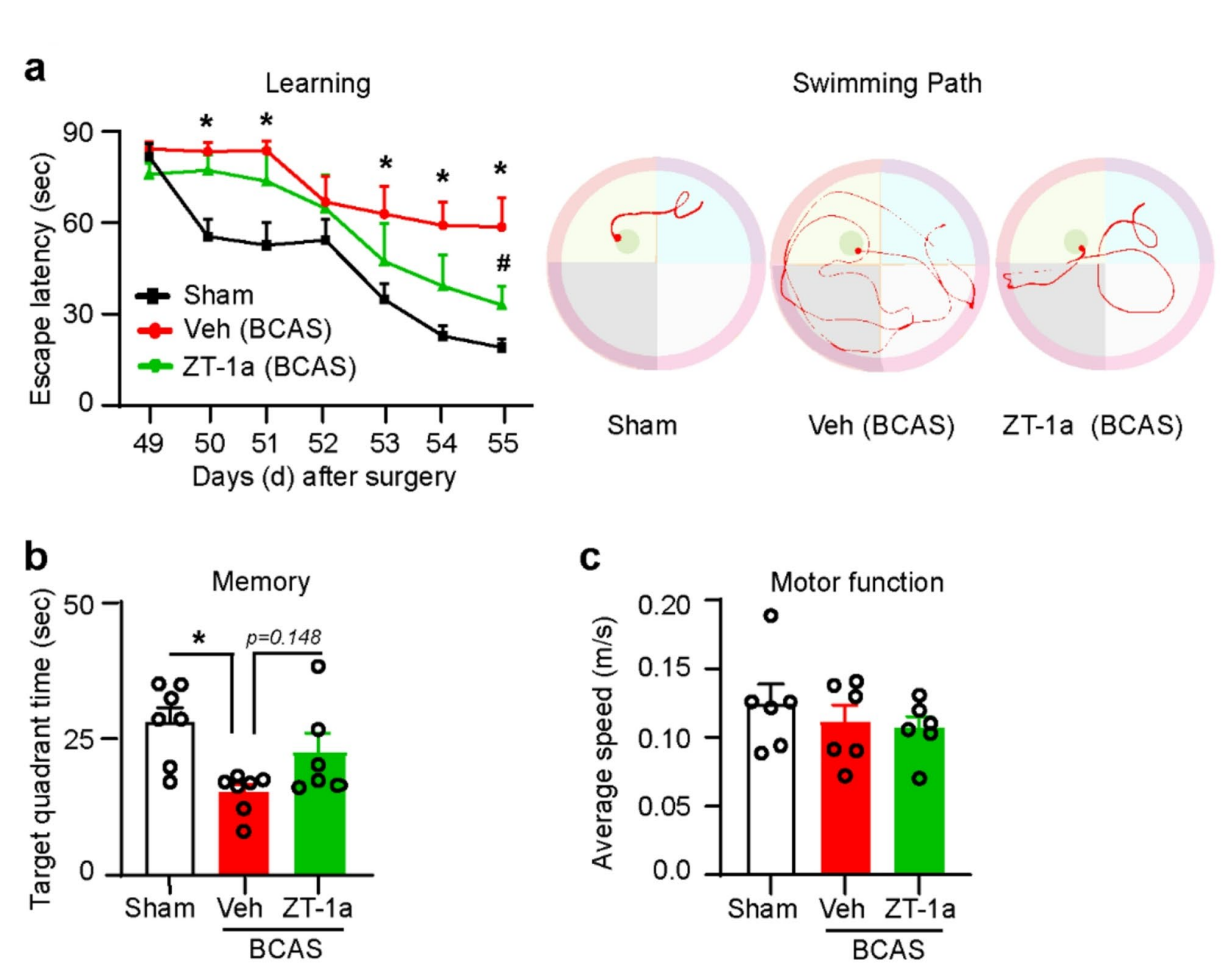
Delayed administration of ZT-1a improves learning and memory function in mice 8 weeks after BCAS. **a** ZT-1a treatment reduces the escape latency times and swimming path compared to Veh-treated BCAS mice during the MWM test for 7 days. Data are mean ± SEM; two-way repeated-measures ANOVA, Tukey’s post hoc test; *n* = 6–7, *p < 0.05, Veh vs. Sham, #*p* < 0.05, Veh vs. ZT-1a. **b** Time spent in the target quadrant during the probe test on day 8 of the MWM test. Data are mean ± SEM; one-way ANOVA, Tukey’s post hoc test; *n* = 6–7, *p < 0.05. **c** Average swimming speed during the probe test on day 8 of the MWM test

Since ZT-1a treatment reversed VCID pathologies (demyelination and neurodegeneration) in BCAS mice, we assessed whether it also associated with cognitive function recovery. The MWM test is commonly used to assess learning and memory in rodents. To evaluate whether ZT-1a treatment can rescue BCAS-induced cognitive impairment, we conducted the MWM test after 7 weeks of BCAS. On the first day of the training for the MWM test, the Sham, Veh-BCAS, and ZT-1a-BCAS groups showed no significant differences in escape latency, as expected given their lack of familiarity with the hidden platform [18]. Compared to the Sham group, Veh-treated BCAS mice exhibited significant spatial learning deficits (*p < 0.05, Fig. 8a) during the 7-day MWM learning task (increased time to reach the platform, 58.12 ± 12.01 vs. 16.12 ± 2.01 s). Importantly, ZT-1a-treated BCAS mice exhibited a significant (#*p* < 0.05) increase in spatial learning (reduced time to reach the platform, 33.07 ± 6.34 s) relative to Veh-treated BCAS mice. In the probe test, ZT-1a-treated BCAS mice spent more time (22.5 ± 3.6 s, Fig. 8b) in the target quadrant compared to Veh-treated mice (15.8 ± 1.2 s), suggesting improved retention of spatial memory. There were no significant differences in motor function, as the average swimming speeds were similar in all groups (Fig. 8c). Collectively, these results suggest that delayed treatment with ZT-1a following BCAS improved learning and memory functions in mice.

## Discussion

An important translational aspect of this study is that ZT-1a treatment during the symptomatic stage of VCID (4–8 weeks post-BCAS surgery) attenuated BCAS-induced pathogenesis, including reactive astrogliosis in mice. At this advanced stage, which mimics human VCID, inhibition of the SPAK-NKCC1 cascade is as effective as in the early stages [9] in reducing BCAS-induced demyelination and memory impairment. A highly novel observation of this study is the biphasic reduction in CBF following BCAS from day 0 to day 56 (Fig. 1c, d), while ZT-1a restores the CBF (Fig. 5a). The biphasic change of CBF, along with the BBB disruption and reactive astrogliosis, highlights the sensitivity of astrocyte modification, particularly vessel-related astrocytic endfeet to pathological conditions such as VCID. These results suggest that astrocyte-specific mechanisms, including the regulation of the SPAK-NKCC1 pathway, may serve as potential modifiable therapeutic targets for improving blood flow and overall brain health in VCID.

The BCAS model induces CCH and replicates key features of VCID, including inflammation, WML, and cognitive deficits, while triggering reactive astrogliosis in white matter, STR, and HP [37]. It is well established that the microcoil-mediated BCAS procedure in mice results in abrupt CBF loss at the onset of BCAS due to sudden stenosis of CCA, and this CBF loss is partially recovered in a month [16]. It was previously believed that one of the limitations of the microcoil BCAS model for studying VCID was its instant reduction in CBF followed by a quick recovery of CBF, which does not accurately reflect the actual VCID process, which develops more slowly and progresses over time in humans [1]. Interestingly, in this study, we observed that CBF loss started again from day 28 post-BCAS (Fig. 1c, d) and was sustained until the endpoint (day 56). While most previous studies have focused on periods up to 4 weeks post-BCAS, we have identified a second, slower reduction in CBF at a later stage (4–8 weeks post-BCAS), making it a suitable model for studying late changes in VCID, including hippocampal neurodegeneration [50]. Since BCAS mice exhibit white matter damage without significant hippocampal injury one month post-BCAS [63], hippocampal degeneration may result from preceding white matter damage. Indeed, in this study, we observed a substantial MBP loss in the white matter tract without any neuronal cell death in the hippocampal CA1 subfield at 4 weeks post-BCAS (data not shown). However, by 8 weeks post-BCAS, a significant neuronal death was detected in the HP (Fig. 7b), despite the initial recovery of CBF (Fig. 1c, d). These data suggest that the memory deficits observed in our study were partly associated with hippocampal neurodegeneration during the late stages of VCID, primarily due to delayed death of CA1, CA2, and CA3 neurons following a second wave of CBF reduction. Our findings, consistent with a previous study, suggest that hippocampal neurodegeneration following BCAS occurs as a delayed response [50]. Although no perfect rodent model for VCID currently exists, we propose that the late-stage changes observed in this microcoil BCAS model provide a valuable approach for studying the long-term effects of CCH, closely resembling the pathophysiology of human VCID.

Astrocytes, alongside neurons, play a crucial role in memory and cognition by regulating synaptic transmission, maintaining neural pathways, and facilitating communication between brain regions essential for learning and memory [38]. Astrocyte response to stimuli or insult is orchestrated by intricate signaling cascades that often involve ion channels and transporters, including NKCC1 [22, 71]. Sodium-dependent ion cotransporter NKCC1 helps maintain chloride homeostasis and regulates potassium and water levels in the brain. NKCC1 is expressed in both neurons and glial cells [31]. NKCC1 expressed in the luminal membrane of the choroid plexus contributes to CSF production [67]. Astrocytic NKCC1 suppresses GABA-mediated excitatory action during seizures, while neuronal NKCC1 has the opposite effect [48], highlighting the importance of NKCC1 as a modifiable target with cell-specific functions. NKCC1 is activated by SPAK kinase, leading to increased cellular stress, inflammation, and disruption of the BBB [84]. Overactivation of the SPAK–NKCC1 complex is well-documented to contribute to ischemic brain damage [51]. NKCC1 upregulation and/or KCC2 downregulation were observed in cerebral stroke [61]. In this study, the colocalization of tNKCC1 with GFAP^+^ astrocytes and, to a lesser extent, Iba1^+^ microglia further support the notion that astrocytes are the primary cell type mediating SPAK-NKCC1 signaling in VCID (Fig. 2c). This is consistent with previous studies showing that astrocytes play a central role in maintaining ion and water homeostasis in the brain and are highly sensitive to ischemic and hypoxic conditions [44]. We found that CCH-induced increased expression and phosphorylation of NKCC1 led to reactive astrogliosis and an increase in the expression of cytotoxic LCN2 (Fig. 4e). A recent study demonstrated that elevated LCN2 levels impair OPC differentiation and maturation, resulting in inadequate remyelination and white matter injury following ischemic insult [35]. Notably, astrocytic LCN2 promotes cell death in OPC, OL, and neurons in rodents [27, 46], and plasma levels of LCN2 were higher in vascular dementia patients compared to healthy control subjects [28]. Therefore, we speculate that reactive astrocyte-secreted LCN2 contributed to OPC, OL, and CA1 neuronal cell death and/or impaired OPC differentiation and maturation, leading to WML and hippocampal neurodegeneration in our study. Importantly, we found that SPAK inhibitor ZT-1a attenuated the transformation of homeostatic astrocytes into cytotoxic reactive astrocytes, as indicated by reduced expression of the proinflammatory markers GFAP, C3d, and LCN2 (Fig. 4d, e), in parallel with reduced OPC, OL, and neuronal cell death (Additional File: Fig. S5 and Fig. 7b). Thus, these findings support our hypothesis that the SPAK-NKCC1 activity contributes to reactive astrogliosis and promotes OL and neuronal cell death, underscoring its potential to mitigate astrocyte-mediated cytotoxicity in VCID. The timing of therapeutic interventions is critical for managing VCID. Previous studies, including our own, have shown that early treatment with NKCC1 inhibitor bumetanide (administered 3–25 days post-BCAS) [79] or SPAK inhibitor ZT-1a (administered 2–4 weeks post-BCAS) can prevent BCAS-induced VCID pathologies, such as astrogliosis, white matter damage, and cognitive decline, demonstrating their preventive efficacy [9]. Here, we show that ZT-1a could be used as a potential curative agent since administration at the advanced stage rescued cognitive impairment by attenuating BCAS-induced VCID pathologies as effectively as early treatment intervention.

CBF autoregulation ensures a stable blood supply and maintains water homeostasis in the brain under normal physiological conditions [67]. Disruptions in CBF have been implicated in various cerebrovascular diseases, including VCID [5, 70]. A recent clinical trial found that lower baseline CBF is associated with the expected mild cognitive decline seen with aging and in patients with VCID [44]. Moreover, reduced CBF has been linked to accelerated cognitive deficits and an increased risk of dementia in the general population [73]. CCH induces several pathological changes in the brain, such as inflammation, WML, neuronal damage, and ultimately, cognitive decline. Therefore, understanding the mechanisms driving CBF reduction and identifying strategies for its restoration are crucial for developing therapeutic approaches to slow or stop VCID progression. A key finding in our study was the secondary and sustained reduction in CBF in mice during the late stage following BCAS (Fig. 1c, d), prompting us to investigate the underlying mechanism of this phenomenon. The neurovascular unit (NVU) consists of cellular (neuron, glia, endothelial, pericyte) and extracellular elements (basement membrane), all of which play a critical role in the dynamic regulation of CBF and the maintenance of BBB function [5, 27]. Astrocytes have a highly complex structure, with multiple branching processes, including endfeet that extensively wrap blood vessels. These endfeet cover 70–100% of the vascular perimeter, isolating brain tissue from the vasculature and facilitating communication between brain cells and blood vessels, orchestrating the CBF [15]. When an end-foot is lost, rapid replacement typically occurs; however, failure to do so leads to BBB disruption [41]. BBB damage can cause neurovascular uncoupling, where the brain’s blood supply and neuronal activity become disconnected, resulting in reduced CBF. In this study, we observed a significant increase in albumin fluorescence intensity surrounding blood vessels in BCAS mouse brains at 8 weeks post-BCAS (Fig. 5b), indicating BBB leakage. BBB disruption allows plasma proteins, such as fibrinogen, to leak into the brain parenchyma, leading to synaptic loss and neuronal dysfunction [47]. It is possible that astrogliosis, neuronal damage, and WML mediated by BBB leakage contributed, at least in part, to the late stage decrease in CBF observed in our study. Additionally, the significant loss of astrocytic endfeet coverage around microvessels in the BCAS mice (Fig. 6e, f) is likely associated with the sustained reduction in CBF. Importantly, ZT-1a treatment prevented BBB damage and reversed the loss of astrocytic endfeet caused by BCAS, supporting our hypothesis that astrocytic NKCC1-mediated inflammation and BBB leakage contribute to the delayed, sustained reduction in CBF in mice.

Astrocytic endfeet al.so ensure ionic and osmotic homeostasis [64] by expressing specialized channels that support their specialized functions, including the AQP4 water channel, the inwardly rectifying K^+^ channel Kir4.1, and the Ca2^+^-dependent K^+^ channel MaxiK [15]. AQP4 plays a key role in astrocytic water fluxes linked to potassium uptake and buffering. Elevated extracellular potassium, whether activity-induced or externally applied, leads to astrocyte swelling and extracellular space shrinkage [46, 72]. Astrocytes maintain a high Cl- concentration with NKCC1, and the resulting electrochemical chloride gradient promotes Cl^−^ efflux [34]. NKCC1 contributes to K^+^ uptake, swelling, and swelling-induced glutamate release in astrocytes in the presence of high extracellular K^+^ [33, 73]. Ionic changes in the perivascular region and astrocyte endfeet proximity might contribute to the observed BBB leakage. Thus, it was possible to reduce this leakage with selective inhibition of SPAK-NKCC1 activity by ZT-1a.

AQP4 anchors to the cell membrane in the astrocyte endfeet by co-expression with dystroglycans. During brain injury β-dystroglycan, which is essential for anchoring proteins at astrocyte endfeet and linking it to the vascular basement membrane, is downregulated along with AQP4, leading to BBB leakage [20]. Moreover, BBB integrity is maintained by TJ proteins, formed by integral membrane proteins like claudins, occludins, and JAMs, along with cytoplasmic accessory proteins such as ZO-1 [25]. Disruption of these TJ proteins increases BBB permeability, which is correlated with reduced CBF and impaired hemodynamics in stroke and AD [30, 60, 74]. While several proteinases can degrade these complexes, MMP-9 is particularly key in degrading dystroglycans and TJ proteins [6]. MMP-9 is highly expressed in astrocytes, which makes it crucial for BBB maintenance [6, 76]. Perivascular neuroinflammation activates MMP-9, which degrades the dystrophin-dystroglycan complex that anchors the astrocytic end-foot to the vascular basement membrane [55]. Endothelial barrier damage correlated with increased MMP-9 activity, which contributes to claudin-5 disruption [66]. In our study, we observed significant upregulation of both MMP-2 and MMP-9 in GFAP^+^ astrocytes in BCAS mouse brains, while ZT-1a treatment notably reduced both MMP-2 and MMP-9 expression (Fig. 6c, d). Our results are consistent with an earlier report showing that upregulation of MMP-2 and MMP-9 causes BBB disruption by degrading TJ proteins in rodent models of VCID [13]. Collectively, in BCAS mice, CBF is reduced by the loss of the astrocytic endfeet from the blood vessels. Several key factors are linked to this event, including disruption in ion and water homeostasis via NKCC1 and AQP4, weakening interactions of the endfeet with the vascular membrane through anchoring proteins and TJ proteins being cleaved by MMP-2 and MMP-9, and oxidative stress. All of these factors work in concert to ultimately reduce the vasodilatation efficacy and prolong CBF reduction.

Brain blood flow can also be increased through neoangiogenesis, which promotes the formation of new functional blood vessels and enhances neurovascular coupling, thereby improving the performance of existing vascular structures [30]. The angiogenesis process is regulated by pro-angiogenic factors such as VEGF and HIF-1α, as well as anti-angiogenic molecules like thrombospondins [54]. Although it is well known that ischemia induces angiogenesis in the brain [83], we found no effect of ZT-1a on angiogenesis in BCAS mouse brains as evidenced by similar levels of VEGF expression and GLUT1^+^ vessel coverage in both Veh and ZT-1a-treated groups (Additional File: Fig. S4b and Fig. 5b). Further, we assessed vascular maturation and found that ZT-1a treatment did not exhibit any significant alterations in vascular maturation marker angiopoietin-1 expression compared to Veh-treated BCAS mice (data not shown). Collectively, these data suggest that the restoration of CBF by ZT-1a treatment is not mediated by an increase in the number of blood vessels or by enhanced vascular maturation. However, a more detailed assessment of vessel volume, length, branching, and tortuosity will be needed to know the precise role of ZT-1a in neoangiogenesis and vascular remodeling.

In this study, we found that astrocytes and microglia are involved in the BCAS-induced disease process, including BBB disruption, CBF loss, and WML, via NKCC1 upregulation. ZT-1a treatment significantly reduces BBB damage and improves CBF. However, we cannot rule out the potential effects of ZT-1a on other brain cell types, including pericytes and endothelial cells, which are key components of the NVU, express SPAK-NKCC1 [31], and play a role in CBF regulation. Pericytes, which are covered by astrocytic endfeet in the capillary basal lamina, are known to be critical for CBF regulation [49]. In our study, we examined pericyte coverage and found no significant difference in the expression of PDGFRβ (a pericyte coverage marker) around blood vessels among the Sham, Veh-treated BCAS, and ZT-1a-treated BCAS groups (Additional File: Fig. S4c), suggesting that the increase in CBF in the mouse brain by ZT-1a is not mediated through improved pericyte coverage. Although our findings suggest that ZT-1a restores CBF by preserving BBB integrity through TJ protein maintenance and enhanced astrocytic endfeet coverage, further studies are needed to elucidate the underlying molecular mechanisms. Calcium signaling in astrocytes exhibits remarkable spatial specificity, advancing our understanding of their complex signaling mechanisms [2, 19]. While this progress has led to significant discoveries, full understanding of astrocytic Ca²⁺ signaling and its implications remains a challenge in this field. A recent study demonstrated that CCH in mice induces a sustained resting intracellular calcium Ca²⁺ influx in astrocytes via activating transient receptor potential ankyrin 1 (TRPA1) ion channel at 14 and 28 days after BCAS [29]. This sustained astrocytic Ca²⁺ influx was accompanied by impaired vasodilation in parenchymal arterioles, leading to reduced responses to low O2 levels and adenosine, which may contribute to the decline in CBF. Whether BCAS-induced prolonged activation of the astrocytic Ca²⁺-TRPA1 pathway contributes to the second phase of CBF reduction observed in this study, and if adenosine changes follow this astrocytic Ca^2+^ deregulation, remains to be determined.

Although our findings provide important insights on VCID pathogenesis, there are several limitations to this study that warrant discussion. First, the SPAK-NKCC1 pathway has several physiological roles, including the regulation of vascular tone and the maintenance of renal ion balance [57, 78], which could become a potential target of the off-site effects of ZT-1a. In the kidney, SPAK regulates the activities of NKCC2 and the NaCl cotransporter (NCC), which are major mediators of NaCl reabsorption in the nephron [58]. Although we used a relatively low dose of ZT-1a (5 mg/kg), prolonged administration could potentially impair NKCC2 and NCC functions, leading to decreased NaCl reabsorption and increased diuresis [3, 11]. Therefore, further studies are needed to assess the safety profile of long-term ZT-1a treatment and its effects on peripheral organs, particularly renal function. Second, our previous findings showed that a 5 mg/kg dose of ZT-1a for day 1 remained effective for up to 7 days post-stroke [82], and administration every 3 days exhibited efficacy for up to 35 days post-BCAS [9]. Therefore, we selected this ZT-1a dose for our current study. Nevertheless, a dose-response study of ZT-1a in the BCAS model is required to fully investigate its therapeutic potential for VCID, which warrants further investigation. Third, we used young adult mice in this study to minimize confounding factors, as they lack comorbidities such as aging, diabetes, or hypertension that can significantly affect cognitive outcomes. However, VCID commonly occurs in older individuals and in the presence of comorbidities. Therefore, future studies in aged or comorbid models will be essential to better define the translational applicability of ZT-1a.

## Conclusion

As of now, there are no FDA-approved treatments for VCID, and current strategies primarily focus on controlling risk factors such as hypertension and diabetes. In this study, we demonstrate that ZT-1a administration during the symptomatic stage of VCID improves learning and memory function by reducing reactive astrogliosis, demyelination, preserving BBB integrity, and restoring CBF in BCAS mice. These findings highlight the therapeutic potential of ZT-1a in counteracting the detrimental effects of cytotoxic reactive astrocytes in VCID and other neurodegenerative disorders associated with BBB dysfunction. Additionally, we present the biphasic CBF reduction and late-stage changes in the BCAS model as potentially unexplored avenues for studying VCID.

## Supplementary Information

Below is the link to the electronic supplementary material.

Supplementary Material 1

## Data Availability

No datasets were generated or analysed during the current study.

## Abbreviations

AD: Alzheimer’s disease
AQP4: Aquaporin-4
BBB: Blood-brain barrier
BCAS: Bilateral common carotid artery stenosis
CA1: Hippocampal CA1 subfield
CBF: Cerebral blood flow
CC: Corpus callosum
CCA: Common carotid artery
CCH: Chronic cerebral hypoperfusion
CTX: Cortex
EC: External capsule
ECA: External carotid artery
GFAP: Glial fibrillary acidic protein
I.P.: Intraperitoneal
ICA: Internal carotid artery
IF: Immunofluorescence
LCN2: Lipocalin-2
MBP: Myelin basic protein
MMPs: Matrix metalloproteinases (MMPs)
MWM: Morris water maze
NVU: Neurovascular unit
OA: Occipital artery
OL: Oligodendrocytes
OPC: Oligodendrocyte progenitor cell
PSD-95: Postsynaptic density protein-95
ROIs: Regions of interest
SPAK: The STE20/SPS1-related proline/alanine-rich kinase
SYP: Synaptophysin
STR: Striatum
TJ: Tight junctions
TL: Tomato lectin
VN: Vagus nerve
WML: White matter lesions
ZO-1: Zonula occludens-1
