# Supplementary material for: Pharmacological inhibition of SPAK-NKCC1 complex attenuates astrogliosis and restores cerebral blood flow in a mouse model of VCID
Source: Acta Neuropathol Commun. 2025 Oct 24;13:216. doi: 10.1186/s40478-025-02137-2 (PMC12551359; doi:10.1186/s40478-025-02137-2)
Supplement: Supplementary file 1 — Supplementary Material 1 [file 40478_2025_2137_MOESM1_ESM.pdf]

Additional file for

Pharmacological inhibition of SPAK-NKCC1 complex attenuates astrogliosis and restores cerebral blood flow in a mouse model of VCID

Khadija Habib[1,2,a], Md Tipu Sultan[1,2,a], Israt Jahan[1,2], Md Shamim Rahman[1], Sujan Kumar Kundu[1], Barnali Sarker[1], Rabia Islam[3], Tanvir Ahmed[1], Ian A. Mendez[2], Guodong Cao[4,5], Dandan Sun[4,5], Vesna Tesic[1,6], Mohammad Iqbal H. Bhuiyan[1,2,6]

Corresponding author

Correspondence to: mohammad.iqbal@lsuhs.edu (Mohammad Iqbal H. Bhuiyan)

This PDF file includes:

Figs. S1 to S6

Table S1

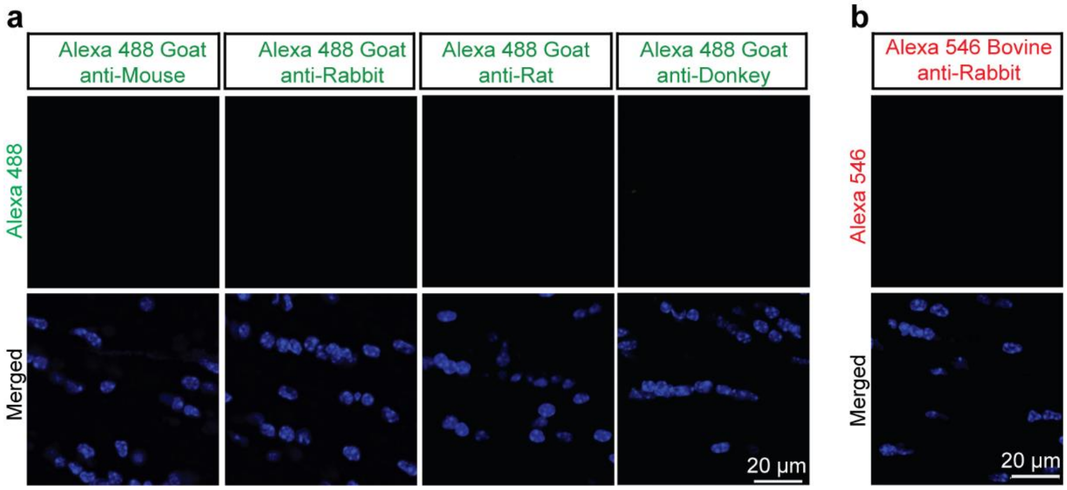

Fig. S1. Detection of immunofluorescence signal with specific secondary antibodies, goat anti-mouse, goat anti-rabbit, goat anti-rat, donkey anti-goat, and bovine anti-rabbit, in mouse brain. (a) Representative confocal images of the Sham brain section immunostained with Alexa Fluor 488 conjugated anti-mouse, anti-rabbit, anti-rat, and anti-donkey secondary antibodies. (b) Immunoassayed images of Alexa Fluor 546-conjugated anti-rabbit secondary antibody-stained brain samples. Nuclei were counterstained with DAPI. Images for all the secondary antibodies were captured using identical confocal microscopy settings, and all the antibodies were used at 1:250 dilutions.

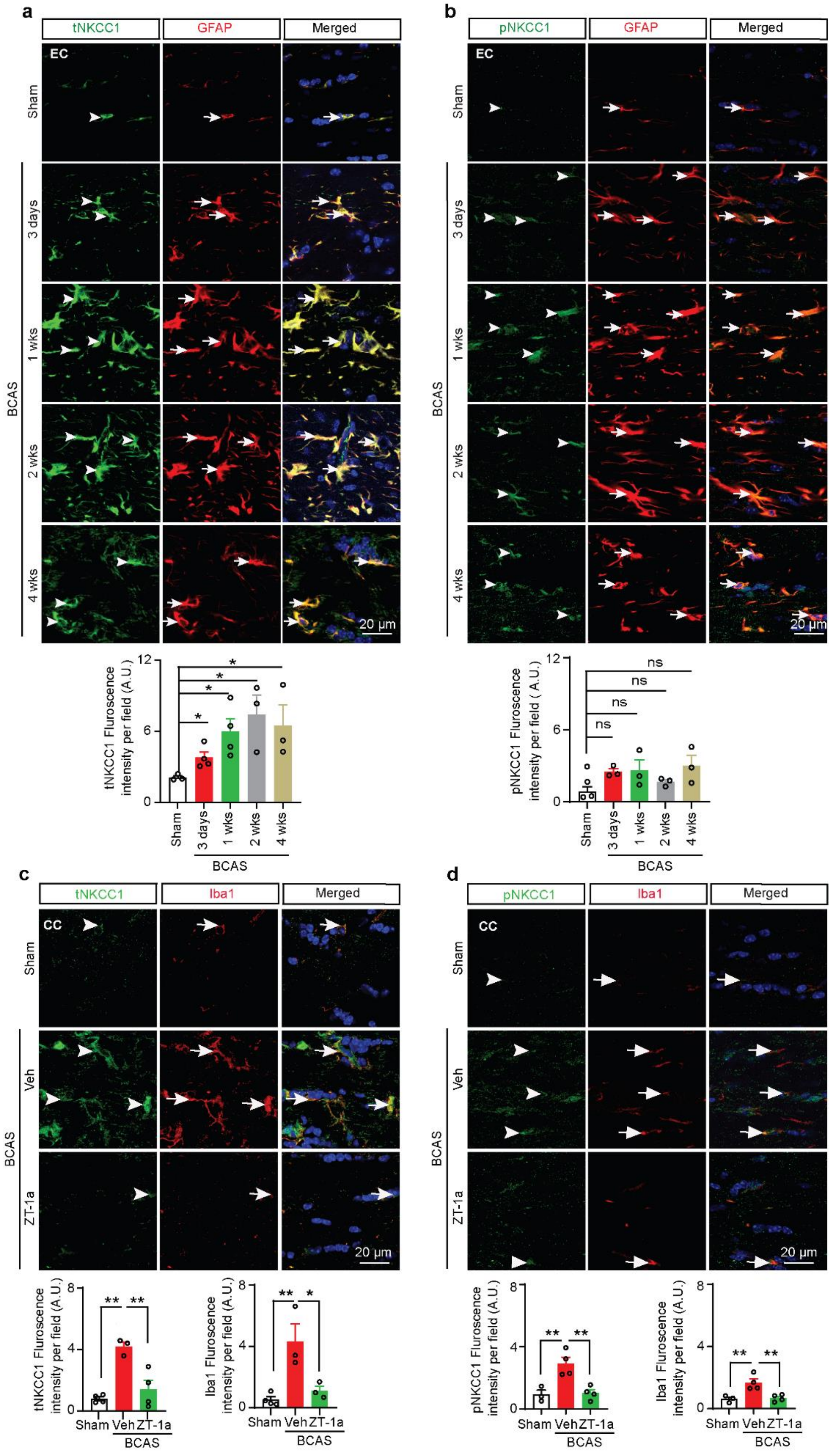

a
tNKCC1
GFAP
Merged
EC
Sham
3 days
1 wks
2 wks
4 wks
BCAS
20 µm
tNKCC1 Fluroscence intensity per field (A.U.)
Sham
3 days
1 wks
2 wks
4 wks
b
pNKCC1
GFAP
Merged
ns
pNKCC1 Fluroscence intensity per field (A.U.)
c
tNKCC1
Iba1
Merged
CC
Veh
ZT-1a
Iba1 Fluroscence intensity per field (A.U.)
d
pNKCC1

Fig. S2. BCAS-induced upregulation of the tNKCC1 and pNKCC1 protein in $GFAP^{+}$ reactive astrocytes and $Iba1^{+}$ microglia/macrophages in white matter. (a) Representative images of the time-dependent expression of tNKCC1 in $GFAP^{+}$ reactive astrocytes in EC of the Sham and BCAS mice brain at 3 days, 1, 2, and 4 weeks after BCAS. Arrowheads and arrows indicate, respectively, tNKCC1 protein and GFAP expressions. Data are mean ± SEM; one-way ANOVA; Tukey's post hoc test; n=3-4, *p<0.05, **p<0.01. (b) Immunostaining analysis of the time-dependent expression of pNKCC1 in $GFAP^{+}$ reactive astrocytes in the EC of Sham and BCAS mice brains at 3 days, 1, 2, and 4 weeks after BCAS. Arrowheads and arrows indicate, respectively, pNKCC1 protein and GFAP expressions. Data are mean ± SEM; one-way ANOVA; Tukey's post hoc test; n=3-4, ns=non-significant. (c-d) Immunostaining analysis of the tNKCC1 and pNKCC1 expression in $Iba1^{+}$ microglia/macrophages in the CC region of the mouse brain at 8 weeks post-BCAS. Arrowheads indicate tNKCC1 (c) and pNKCC1 (d) expressions. Arrows indicate Iba1 expressions (c-d). Data are mean ± SEM; one-way ANOVA; Tukey's post hoc test; n=3-4, *p<0.05, **p<0.01.

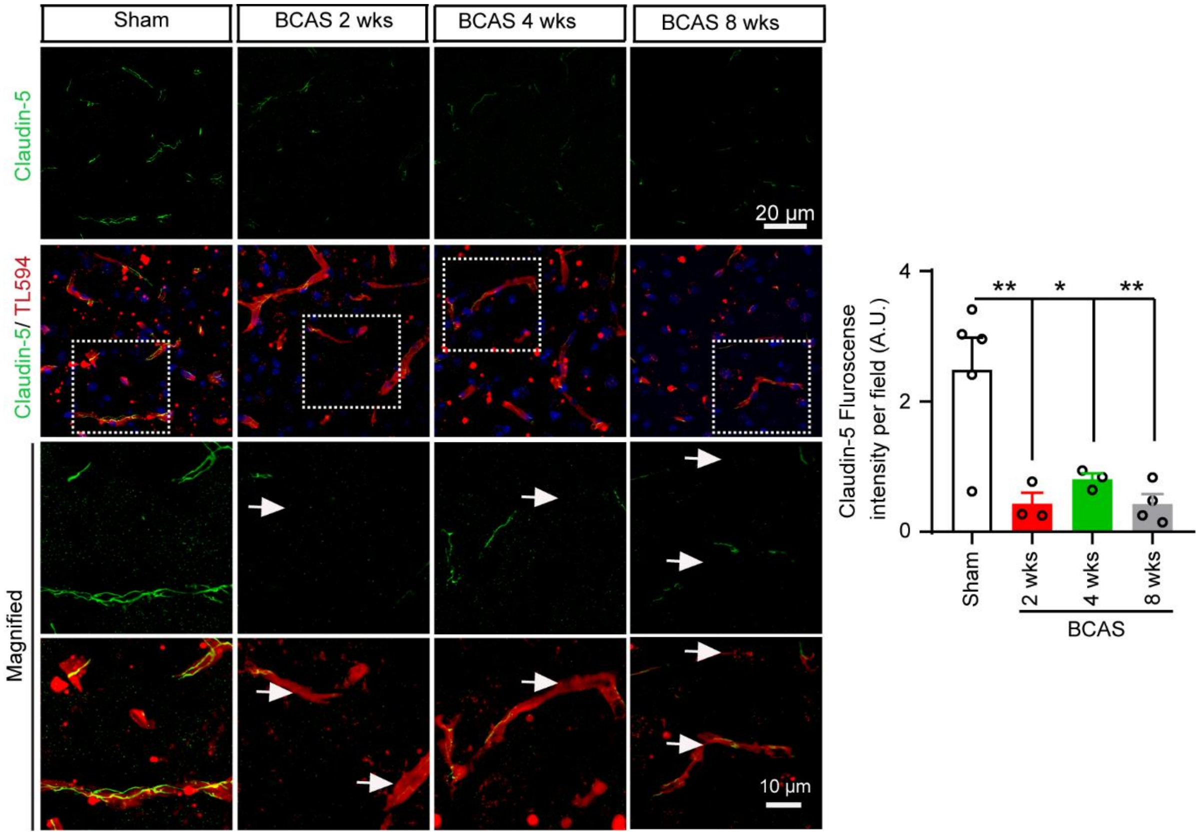

Fig. S3. BCAS induces sustained BBB damage in the subcortical area of mouse brains. Representative images of Claudin-5 and Tomato lectin immunostaining analysis in the cortex of Sham and BCAS mice brains at 2, 4, and 8 weeks after BCAS surgery. The arrow indicates Claudin-5 expression. Data are mean ± SEM; one-way ANOVA; Tukey's post hoc test; n=3-4, *p<0.05, **p<0.01.

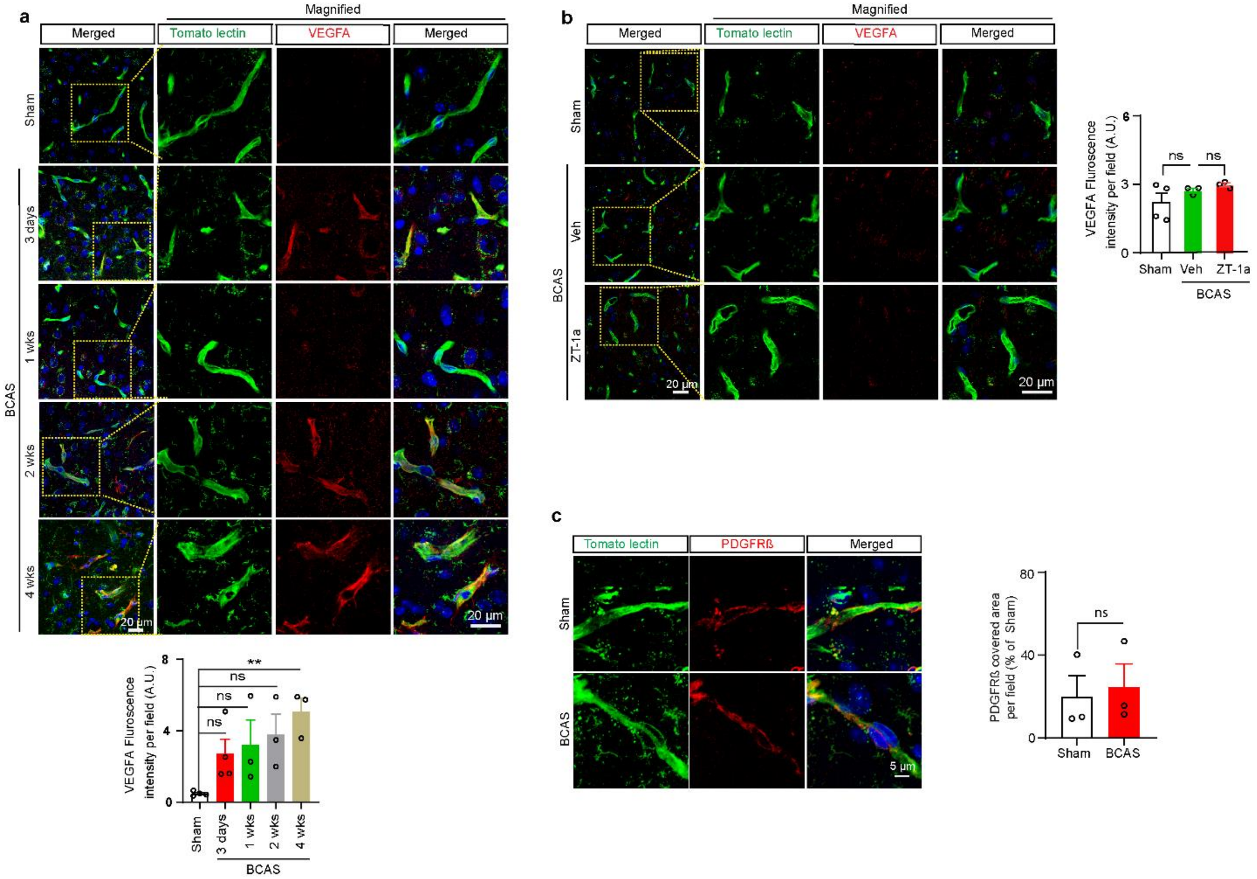

Fig. S4. SPAK inhibition with ZT-1a does not affect angiogenesis, and pericyte coverage does not play any significant role in BCAS-induced CBF loss in the mice brains. (a) Representative images of VEGFA and Tomato lectin immunostaining analysis in the cortex of Sham and BCAS mice brains at 3 days, 1 week, 2 weeks, and 4 weeks after BCAS. Data are mean ± SEM; one-way ANOVA; n = 3-4. (b) Immunostaining analysis of VEGFA and Tomato lectin in the cortex of Sham, Veh-, and ZT-1a-treated mice at 8 weeks after BCAS. Data are mean ± SEM; one-way ANOVA; n = 3-4, ns = non-significant. (c) Representative images of PDGFRβ and Tomato lectin immunostaining analysis in the cortex of Sham and BCAS mice brains at 8 weeks post-BCAS surgery. Data are mean ± SEM; one-way ANOVA; n = 3, ns = non-significant.

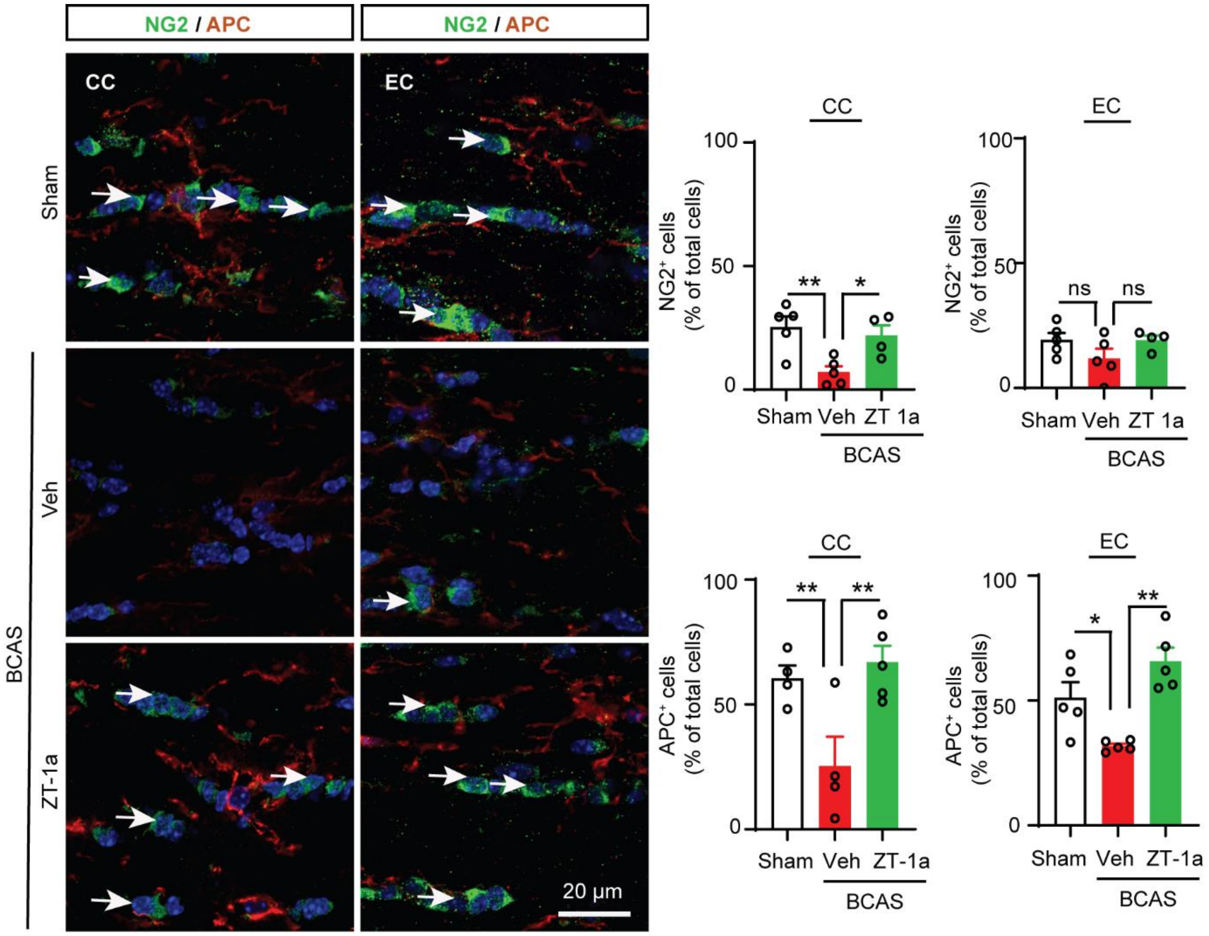

Fig. S5. Immunofluorescence analysis of NG2/APC in the CC and EC regions of mouse brains at 8 weeks post-BCAS. Arrows indicate NG2 expression. Quantitative analysis; Data are mean ± SEM, one-way ANOVA, Tukey's post-hoc test; n=3-5, $*p < 0.05$, $**p < 0.01$.

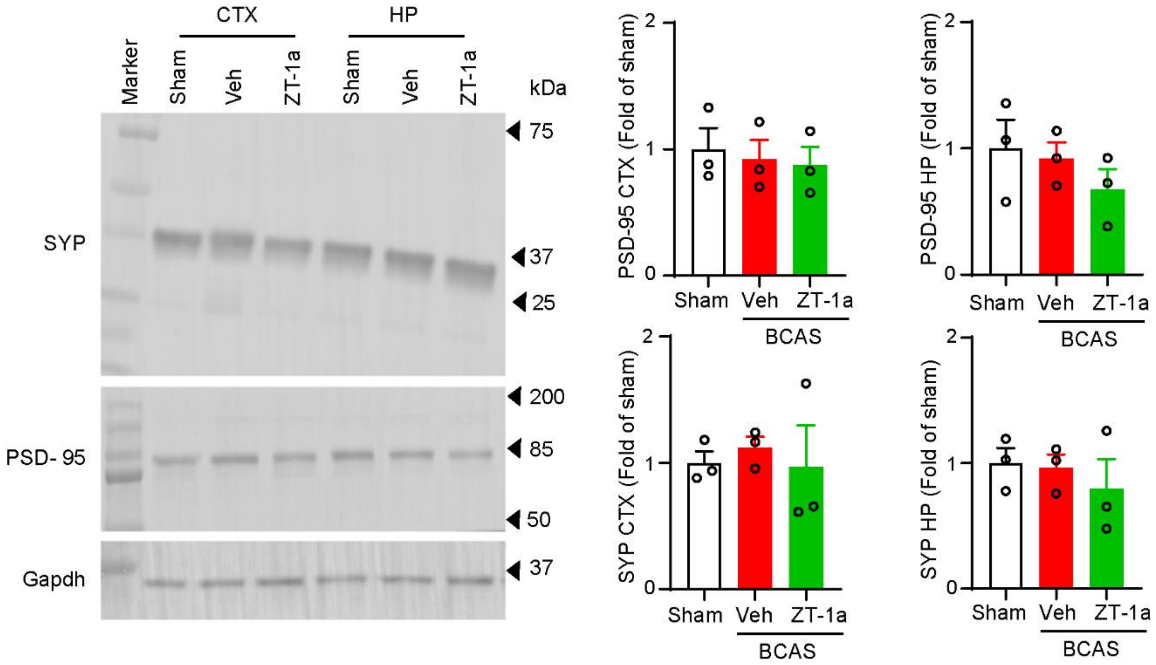

Fig. S6. BCAS does not change synaptic protein expression in the cortex (CTX) and hippocampus (HP) regions of the mouse brain after 8 weeks of surgery. Western blot (WB) analysis shows no changes in synaptic markers SYP and PSD-95 in Sham-operated, Veh-, and ZT-1a- treated mouse brain at 8 weeks post-BCAS. Quantitative analysis; Data are mean ± SEM, one-way ANOVA, Tukey's post-hoc test; n=3.

Table S1. List of antibodies used in this study for immunofluorescence (IF) and Western blot (WB) analysis.

Antibody	Host	Dilution	Company	Catalog No.	Application
tNKCC1	Mouse	1:100	DSHB	T4	IF
pNKCC1	Rabbit	1:200	EMD Millipore	ABS1004	
GFAP	Rabbit	1:100	Agilent Dako	Z0334	
Iba-1	Rabbit	1:200	FUJIFILM	NC9288364	
Iba-1	Mouse	1:100	Millipore Sigma	MABN92	
NG-2	Rabbit	1:200	EMD Millipore	AB5320	
APC	Mouse	1:100	Millipore Sigma CALBIOCHEM	OP80	
NeuN	Rabbit	1:500	Abcam	AB177487	
C3d	Goat	1:100	R&D system	AF2655	
Claudin-5	Rabbit	1:100	Thermo Fisher Scientific	34-1600	
Albumin	Rabbit	1:200	Abcam	AB19196	
VEGFA	Rabbit	1:200	Abcam	Ab52917	
MBP	Rabbit	1:500	Abcam	ab40390	
SMI32	Mouse	1:1000	BioLegend	801701	
GFAP	Mouse	1:200	Cell Signaling	3670S	
LCN2	Rat	1:100	Abcam	ab70287	
AQP4	Rabbit	1:100	Proteintech	16473-1AP	
MMP2	Rabbit	1:100	Proteintech	10373-2-AP	
MMP9	Rabbit	1:100	Proteintech	10375-2-AP	
ZO-1	Rabbit	1:100	Thermo Fisher Scientific	617300	
GLUT1	Mouse	1:100	Abcam	AB40084	
Tomato lectin 488		1:100	VectorLabs	DL-1174	
Tomato lectin 594		1:100	VectorLabs	DL-1177	
SYP	Mouse	1:8000	Santa Cruz	sc-17750	WB
PSD-95	Rabbit	1:2000	Abcam	ab18258	WB
